# Surface Modification of Polyesters Using Biosourced Soil-Release Polymers

**DOI:** 10.1021/jacsau.4c00908

**Published:** 2025-02-05

**Authors:** Matthieu Starck, Emanuella F. Fiandra, Josephine Binks, Gang Si, Ruth Chilton, Mark Sivik, Richard L. Thompson, Jing Li, Mark R. Wilson, Clare S. Mahon

**Affiliations:** † Department of Chemistry, Durham University, Durham, DH1 3LE, United Kingdom; ‡ The Procter & Gamble Newcastle Innovation Centre, Whitley Road, Newcastle upon Tyne NE12 9BZ, United Kingdom; § Procter & Gamble Company, Fabric & Home Care Innovation Centre, Cincinnati, Ohio 45217, United States

**Keywords:** polymers, surface modification, soil-release polymers, biosourced monomers, detergent formulation

## Abstract

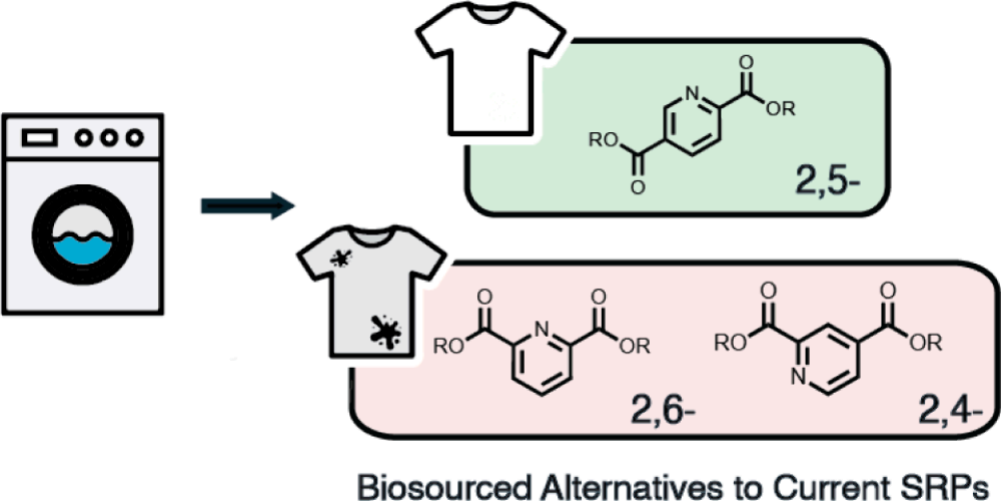

Soil-release polymers (SRPs) are important components of fabric care formulations, performing important roles in the cleaning of synthetic fabrics. SRPs modify the surface of textiles and render materials resistant to staining, while offering environmental benefits by enabling effective cleaning using shorter, cooler wash cycles. Most SRPs used in formulations contain petroleum-sourced terephthalic acid, limiting the environmental benefits presented by the use of these key additives. Here, we have prepared SRPs using a selection of pyridine dicarboxylate monomers that can be accessed from biomass and assessed their ability to modify polyester surfaces. Interestingly, a wide range of surface deposition behavior was observed, with soil-release performance significantly impacted by the pyridine dicarboxylate component in use. The performance of polymers containing 2,5-pyridine dicarboxylate units exceeded or was comparable to that of current industry-standard SRPs, while polymers constructed using 2,4- or 2,6-pyridine dicarboxylate units displayed poor performance. Through a range of studies including dynamic light scattering, contact angle analysis, scanning electron microscopy, and molecular modeling we have explored the solution and interfacial behavior of SRPs and propose the observed changes in performance to arise from a combination of differences in solution self-assembly and variation in affinities for polyester surfaces. Our work highlights the potential of using biosourced starting materials in the replacement of petroleum-derived polymers within formulated consumer products and presents a rationale for the design of SRPs.

## Introduction

Modifying the surface of a polymer can impart properties or behavior at interfaces which differs from the bulk material, an approach which can be used to tune surface energies, or modulate properties such as adhesion or wetting.^1,2^ This concept has found application in a range of areas including electronics, microfluidics and biomedical applications.^3−6^ One area where surface modification of polymer materials has found useful commercial application is in fabric care formulations, particularly within laundry detergents, which contain a range of polymeric components which underpin their function. In addition to performing functions in dispersion, polymer components can be designed to deposit on the surfaces of textiles, altering the surface properties to improve the appearance or texture of the fabric. Soil-release polymers (SRPs) deposit on the surface of textiles including polyethylene terephthalate (PET), rendering the surface hydrophilic and therefore resistant to the deposition of hydrophobic contaminants, termed ‘soil,’ which are suspended in the wash liquor. This change in the surface polarity additionally assists with subsequent cleaning of the fabric, enabling effective removal of soil at lower wash temperatures, shorter wash cycles and with reduced quantities of water. The environmental impacts of such changes can be profound–effective cleaning in cold water presents the largest opportunity to reduce domestic indirect greenhouse gas emission in the fast-moving consumer goods cleaning sector.^7^ Here, SRPs can play an important role in reducing the environmental footprint of cleaning textiles: washing at 30 °C rather than 40 °C can reduce energy consumption by 40% per cycle,^8^ offering significant reductions given that annual electrical consumption on laundry per household exceeds 100 kWh in Europe and North America.^9^ Using shorter wash cycles additionally presents the opportunity to reduce usage of water, an increasingly scarce resource, to help meet a globally sustainable target consumption of 50 L/person/day.^10^ The contribution of laundry to overall water usage is significant: in 2013, an estimated 19 bn m^3^ water was used by 840 m domestic washing machines worldwide.^11^

**Figure 1:**
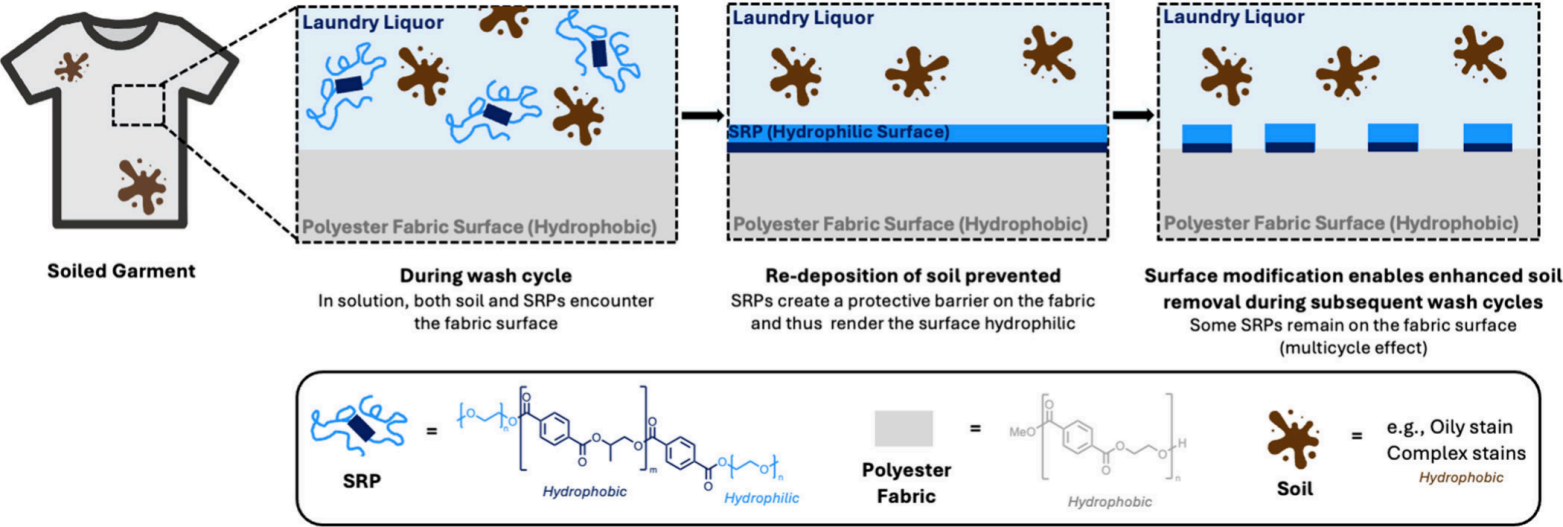
Soil-release polymers (SRPs) deposit on fabric surfaces, promoted by interactions of the hydrophobic block with the surface, and thus render the surface hydrophilic due to the exposed PEG blocks on the surface, preventing the redeposition of soil during the wash and enhancing its removal during subsequent wash cycles.

While presenting clear environmental benefits in terms of enabling cleaning at decreased energy cost and water usage, the environmental impact of additives used in detergent formulations should not be ignored. A common class of commercially used SRPs^12^ comprises (Figure 1) of a poly(ethylene) or (propylene) terephthalate block or blocks, which can adhere to the surfaces of polyester fabrics, and polyethylene glycol (PEG) blocks which extend from the interface, rendering the surface hydrophilic. A key raw material for the production of these polymers, terephthalic acid, is largely produced via the catalytic oxidation of petroleum-sourced *p*-xylene,^13^ presenting opportunities for its replacement with biosourced alternative monomers,^14^ including aromatic diacids such as furan-2,5-dicarboxylic acid,^15,16^ which can be sourced from lignocellulosic biomass. This monomer unit has already been used to produce polymers with similar properties to PET,^17,18^ which may find application in packaging films,^19^ or in the production of alternative textiles.^20,21^ Beyond furan-2,5-dicarboxylic acid, lignocellulosic biomass^22^ presents significant opportunities for the replacement of traditional petroleum-derived building blocks such as terephthalic acid. Lignocellulosic biomass can be rapidly produced at low cost,^23^ or obtained from forestry or agricultural waste, at no detriment to the production of foods.^24^

Here, we present A(B–C)A triblock SRPs containing pyridine dicarboxylate monomers^25,26^ (**1**-**3**) as an alternative aromatic unit to terephthalate. These biosourced building blocks may be accessed through the fermentation of lignin^27^ or produced in engineered *Escherichia coli*,^28^ presenting a promising alternative to petroleum-derived, terephthalate-based SRPs currently in use. In addition to presenting a more sustainable route to the production of these key additives, the use of biosourced aromatic diacids also presents an opportunity to explore the effects of structural isomerism within the aromatic unit, as multiple pyridine dicarboxylate isomers may be sourced from biomass. Interestingly, markedly different performance is observed depending on the isomer of pyridine dicarboxylate used to construct the central block, with 2,5-pyridine dicarboxylates displaying significantly improved performance compared to 2,4 and 2,6-isomers, and 2,5-pyridine dicarboxylate-terephthalate copolymers displaying further enhanced behavior. These differences in behavior have been explored using dynamic light scattering (DLS), contact angle measurements and scanning electron microscopy (SEM), in addition to molecular modeling approaches. This work provides a framework for the rational design of new, more environmentally friendly SRPs using biosourced starting materials.

## Results and Discussion

### Polymer Synthesis

**Scheme 1:**
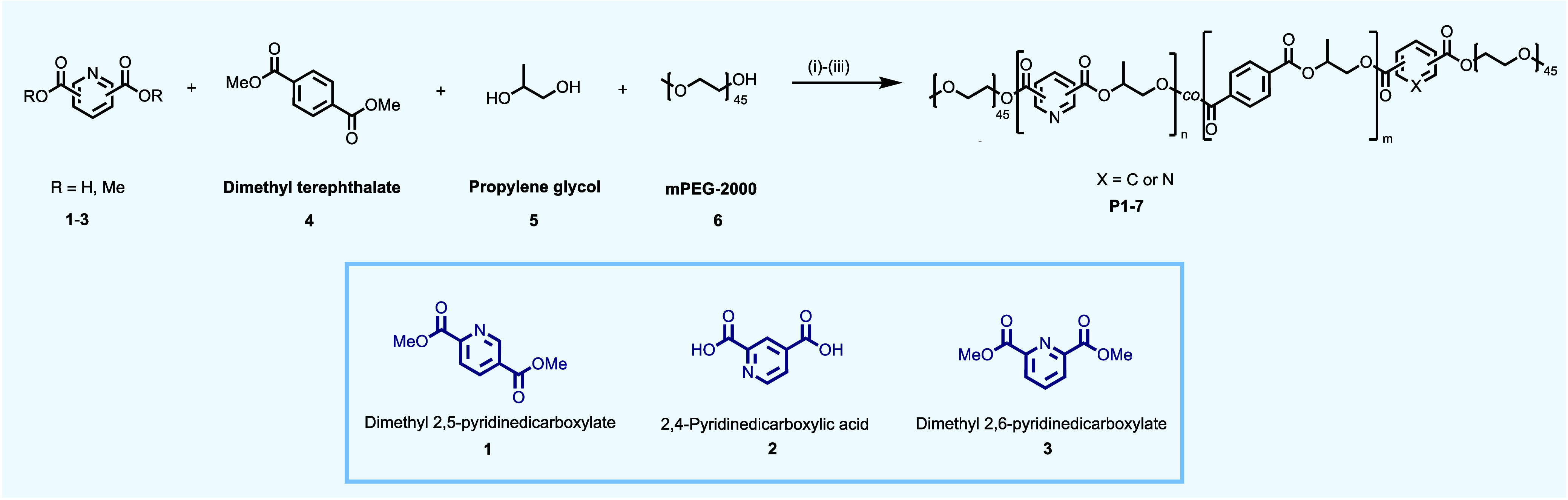
General Synthesis of SRPs in a One-Pot Polycondensation Reaction Conditions: (i) 170 °C, Ar, 2 h; (ii) 210 °C, Ar, 1 h; (iii) 210 °C, 1 mbar, 3 h.

A series of poly(propylene pyridine dicarboxylate)-PEG triblock copolymers (**P1**-**P6**), a pyridine dicarboxylate-terephthalate copolymer (**P7**), and a poly(propylene terephthalate)-PEG triblock reference copolymer (**P8**) were prepared via a one-pot, multistep polycondensation reaction (Scheme 1), following a protocol adapted from the literature.^29,30^ Briefly, initial transesterification between an aromatic diacid or diester (**1**-**4**) and propylene glycol (**5**) generates primarily a diester intermediate. Subsequent polycondensation, at elevated temperature and under reduced pressure, generates the A(B–C)A triblock polymer (**P1**-**P8**), capped with a 2 kDa polyethylene glycol monomethyl ether component (**mPEG-2000, 6**) at both chain termini. A series of triblock polymers of varied monomer composition were prepared using this methodology (Table 1).

**Table 1:** Synthesis and Structural Parameters of Pyridine Dicarboxylate Based SRPs, **P1**–**P7**, and Reference SRP **P8**

	Pyridine dicarboxylate											
Polymer	**1**/eq.	**2**/eq.	**3**/eq.	**4**/eq.	**5**/eq.	**6**/eq.	n	m	*M*_n_^a^/g mol^–1^	*M*_n_^b^/g mol^–1^	*M*_w_^b^/g mol^–1^	Đ^b^
**P1**	10	-	-	-	400	2	6	0	5400	1100	2800	2.5
**P2**	20	-	-	-	400	2	10	0	6200	1200	3500	2.9
**P3**	-	10	-	-	400	2	6	0	5400	1200	3400	2.8
**P4**	-	20	-	-	400	2	10	0	6200	910	2600	3.2
**P5**	-	-	10	-	400	2	6	0	5400	870	2300	2.6
**P6**	-	-	20	-	400	2	10	0	6200	790	2300	2.9
**P7**	4	-	-	10	400	2	3	6	6000	790	2800	3.5
**P8**	-	-	-	10	400	2	0	5	5190	1200	3300	2.8

a As determined by ^1^H NMR spectroscopy.

b As determined by gel permeation chromatography in 0.01 M NaNO_3(aq)_ (1.0 mL/min), calibrated against near-monodisperse PEO standards

### Performance Studies: Anti-redeposition Properties

**Figure 2:**
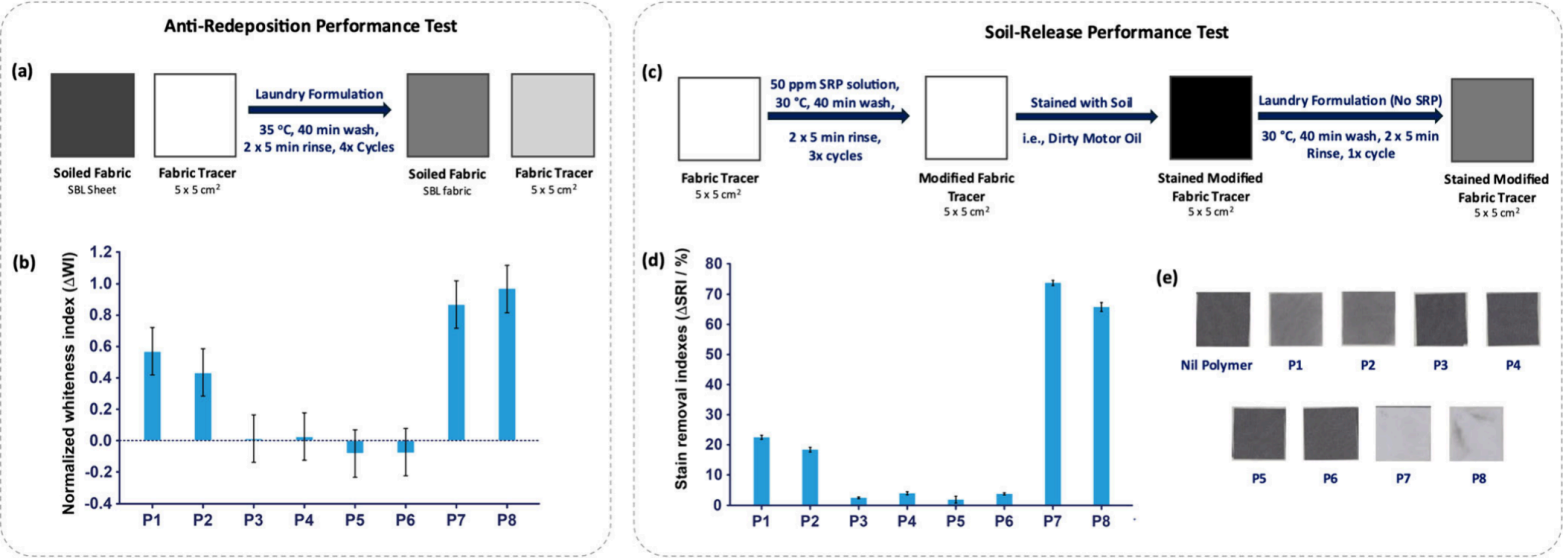
(a) Anti-redeposition test conducted under global wash conditions. Figure adapted from ref (32). Copyright 2022 American Chemical Society. (b) Difference in whiteness index variation (ΔWI) of polyester tracers washed with a laundry detergent formulation with 1% (w/w) SRP. The baseline 0.0 indicates the performance of the SRP-free negative control (Nil). (c) Soil-release performance test conducted under global wash conditions. Figure adapted from ref (32). Copyright 2022 American Chemical Society. (d) Difference in stain removal index (ΔSRI) values obtained for SRPs **P1** to **P8**. (e) Photographs of fabric surfaces captured during soil-release performance testing.

The ability of **P1**-**P7** to modify the surfaces of fabrics and prevent soil redeposition in a representative laundry formulation was initially investigated using anti-redeposition performance tests. Here, SRPs are evaluated for their ability to prevent redeposition of suspended soil, transferred from a soiled fabric swatch, onto white fabric tracers during the wash process (Figure 2a). Redeposition during a wash cycle can lead to the surface of the white fabric tracer appearing gray. CIELAB color space chromaticity coordinates^31^ L_n_*, a_n_* and b_n_* are measured for fabric tracers before and after washing under standard D65 illumination. The whiteness index (WI), as defined by the International Commission on Illumination (CIE), can be calculated using eq 1:^31^
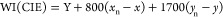
1where Y represents the luminance factor of the light source; *x*_n_ and *y*_n_ are the chromaticity coordinates for the CIE standard illuminant and source used; *x* and *y* are the chromaticity coordinates of the specimen under investigation.

This anti-redeposition test was conducted using a high throughput tergotometer system, washing for 40 min at 35 °C followed by two 5 min rinses, using medium-hard water (∼21 grains per gallon; gpg). Commercially sourced artificial soil sheet (SBL2004 WFK, Krefeld, Germany) was cut into 5 × 5 cm^2^ squares and included in each wash. together with white polyester fabric tracers (also 5 × 5 cm^2^ squares). Overall, four washing cycles were completed, with the soiled fabric sheets replaced after each cycle (SI Section 3.1).^32^ Image analysis was then used to quantify antiredeposition performance in terms of the change in whiteness index (ΔWI) of white polyester fabric tracers which were treated with SRPs under conditions that mimic the laundry environment. The performance of **P1**-**P7** was evaluated against that of the terephthalate-based reference polymer **P8**, which represents a commercially available SRP used in detergent formulations. A negative control (Nil) was performed, in which no SRPs were present in the detergent formulation, to allow a direct comparison with detergents formulated with SRP (1% w/w). The difference in whiteness index, ΔWI, was therefore determined between fabric tracers washed with SRP-containing formulations and the negative control. SRPs that were found to perform well display a high positive ΔWI value, signifying a high soil antiredeposition performance.

The 2,5-pyridine dicarboxylate-based polymers (**P1**, **P2**) were found to display good anti-redeposition performance on polyester (Figure 2b), with whiteness indexes ΔWI ≈ 0.6 and 0.4 respectively; normalized against ΔWI = 1.0 for the reference SRP **P8**. Interestingly, the 2,4-pyridine dicarboxylate series (**P3**, **P4**) and 2,6-pyridine dicarboxylate series (**P5**, **P6**) did not show favorable performance, with ΔWI ≈ 0. These observations suggest that isomerism within the aromatic dicarboxylate unit of the polymers can significantly affect antiredeposition performance, with the underlying cause of these differences not immediately apparent. A copolymer containing 3 units of 2,5-pyridine dicarboxylate and 6 units of terephthalate in the central region, **P7**, was found to display equivalent antiredeposition performance to **P8**, suggesting that current antiredeposition performance levels could be achieved with SRPs containing significant quantities of biosourced starting materials.

### Performance Studies: Soil-Release Behavior

Having identified the 2,5-pyridine dicarboxylate based polymers **P1** and **P2** as promising biosourced alternatives to terephthalate-based SRPs in terms of anti-redeposition performance, their ability to modify the surface of polyester fabric was further investigated in a soil-release performance test. Here, polyester swatches were preconditioned with a solution of SRP by washing in the high-throughput tergotometer for 40 min at 30 °C, with two 5 min rinses, using medium-hard water (8 gpg). An experiment where swatches are washed only with water was included as a negative reference. The tracers were dried and then treated with dirty motor oil before undergoing a further wash cycle under the same conditions with a detergent formulation that does not contain SRP (Figure 2c; SI Section 3.2). This allows assessment of the effects of pretreating the fabric surface with SRP on stain removal in the subsequent wash phase. A reflection spectrophotometer (DigiEye) was used to acquire images of fabrics before and after washing against a white background, and images were analyzed using DigiEye software. For each tracer the color of the motor oil stains was measured by reading the coordinates L*, a*, and b* defined in the CIELAB color system of the stained area itself and the clean background fabric. From the measured coordinates the differences in lightness (ΔL_n_*), redness (Δa_n_*), and blueness (Δb_n_*) in contrast to the unstained background area was calculated. The relative color changes, ΔE*, were calculated to determine the level of staining compared to the unsoiled fabric (eq 3), where the suffix 1 denotes the values for the unsoiled background fabric prior to washing, and the suffix 2 denotes the values for the stain either before or after washing. ΔE* was calculated for both unwashed (A) and washed stains (B). The stain removal index (SRI) was calculated using ΔE* values for unwashed stains and washed stains (eq 4).^32^


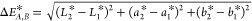
3
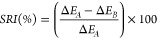
4

Polymers constructed using the 2,5-pyridine dicarboxylate monomer **1**, **P1** and **P2**, displayed favorable performance, with enhanced stain removal from the surface observed compared to fabrics pretreated with water instead of SRP solution (Figure 2d-e). In line with our observations related to antireposition performance, **P3**-**P6** did not show any favorable behavior, with ΔSRI values <10% in each case. The copolymer **P7** displayed improved performance compared to the reference SRP **P8**, demonstrating that introduction of a proportion of **1** can enhance the soil-release behavior of terephthalate-based SRPs, in addition to improving the environmental footprint of the material.

The differences in the performance of polymers constructed using different isomers of the pyridine dicarboxylate units could not immediately be rationalized. Hence, we performed a range of experiments to probe both the surface activity and solution behavior of SRPs using simplified representative systems to establish the factors contributing to differences in soil-release and anti-redeposition performance, and to better understand the mechanism of surface deposition and modification of polyester surfaces using SRPs.

### Surface Modification

**Figure 3:**
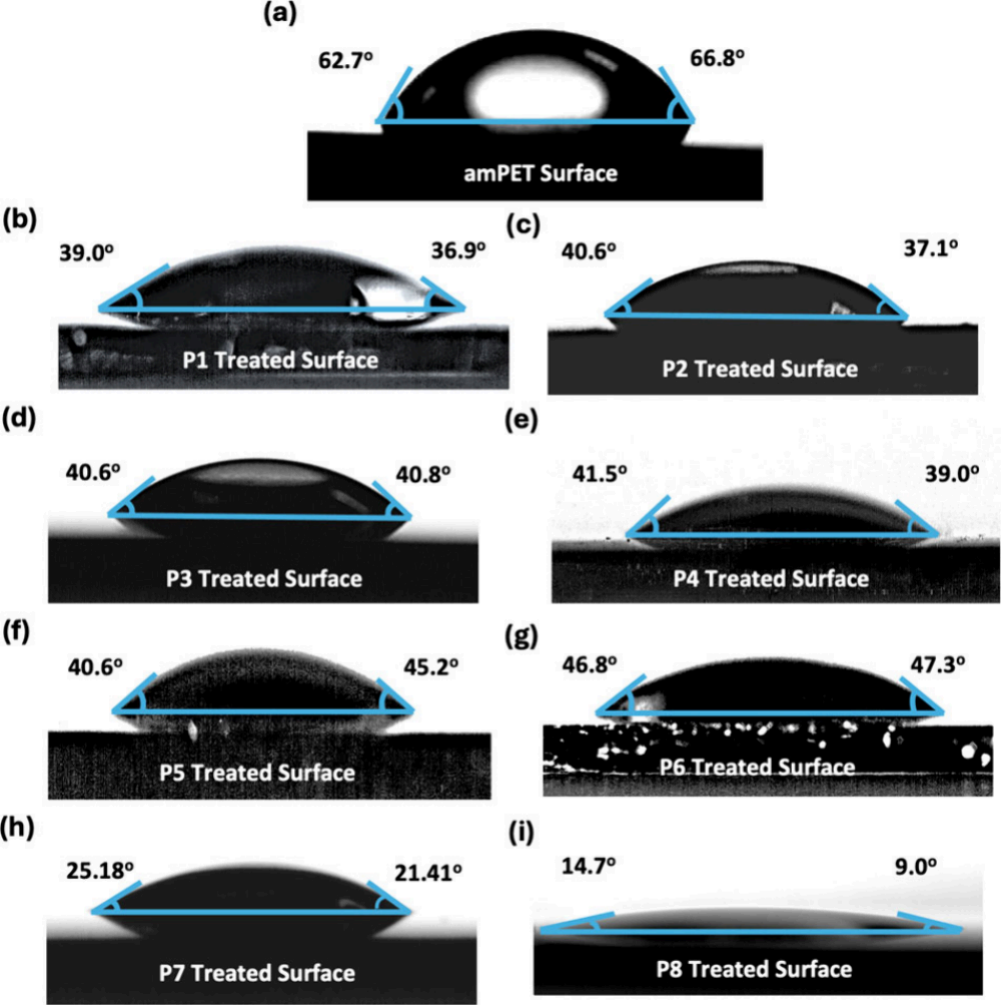
Measured contact angles of a 5 μL droplet of deionized water on an amPET surface and amPET surfaces treated with SRPs (1% (w/w)): (a) Untreated surface, (b–i) SRP-modified surface.

Our initial hypothesis was that differences in soil-release performances exhibited by polymers containing different structural isomers of the pyridine dicarboxylate unit were likely due to differences in surface adsorption i.e. those polymers that performed favorably are deposited onto fabric surfaces to a greater extent than poorly performing polymers. To investigate the extent to which **P1**-**P7** modify the surface of polyester, compared to the reference polymer, **P8**, PET surfaces were generated by spin-coating a solution of amorphous PET (amPET, 1% w/w in CHCl_3_) onto silicon wafer (2000 rpm, 30 s). Surfaces were treated with solutions of **P1**-**P8** (1% w/w) for 40 min, and left to dry upside down to allow excess SRP to run off the surface. A 5 μL droplet of deionized water was then placed on each of the polymer-treated amPET surfaces and the contact angle was measured at room temperature (Figure 3). An average contact angle of 65° was measured for a droplet of water on a neat amPET surface, which was reduced to around 40° for surfaces modified with **P1** to **P5**, rendering the surface more hydrophilic. Surfaces treated with **P6** also followed this trend but to a lower extent, with an average contact angle of 47° measured. Interestingly, treatment with **P7** was found to yield surfaces with a significantly lower average contact angle of 23°, an observation that correlates with the favorable performance of **P7** in antiredeposition and soil release test, while remaining higher than that of a surface treated with reference polymer **P8**, which displayed an average contact angle of 12°. The reductions in water contact angles observed after treatment of PET surfaces with **P1**-**P7** suggest that all polymers tested do render the surface hydrophilic upon deposition, and that variation in performance may partially arise from differences in the extent of the deposition of each polymer on the surface.

**Figure 4:**
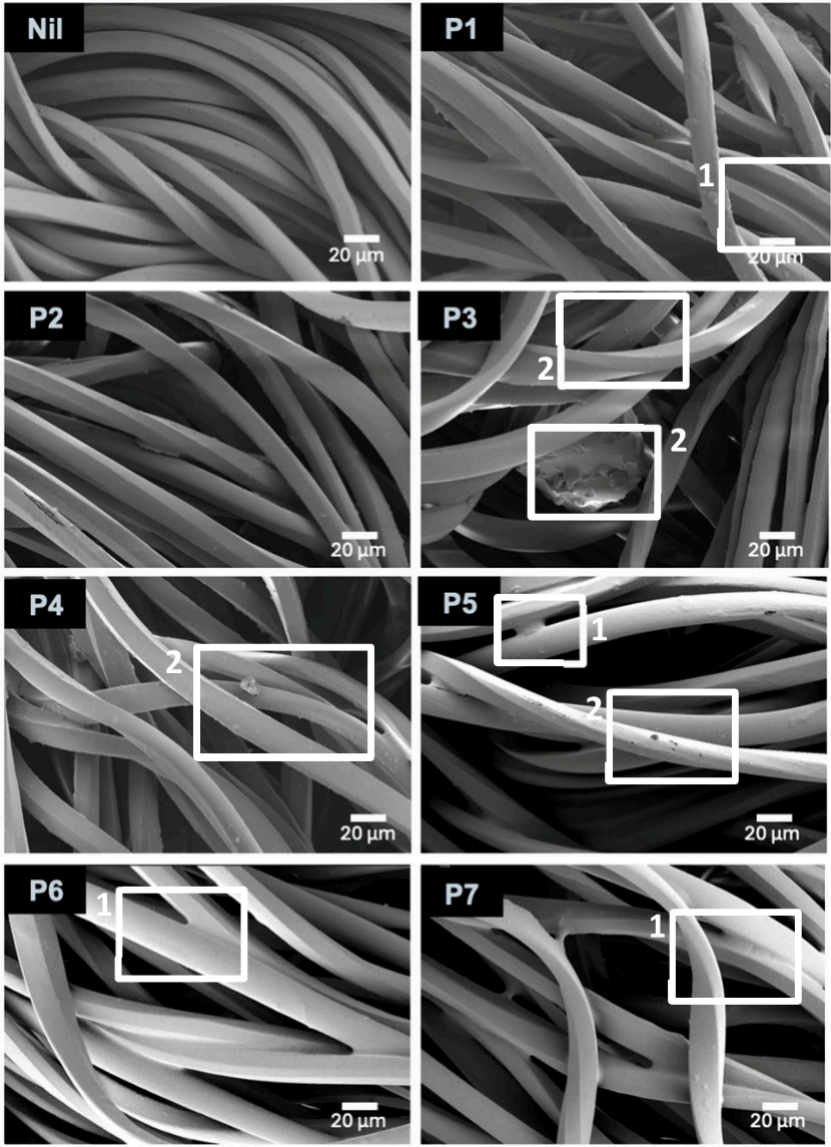
SEM images of polyester (Nil) and SRP-modified fabrics (**P1** to **P7**) with a gold–palladium sputter coating thickness of around 38 nm; SEM images taken at a magnification of 500×. Some surface features have been highlighted: 1. SRPs appearing to deposit between fibers; 2. irregular deposits on fibers.

To further investigate the surface deposition behavior of **P1**-**P7**, SEM imaging was performed to investigate the morphological changes of the polyester fibers induced by the deposition of SRPs. Samples for image analysis were prepared by soaking 1 × 1 cm^2^ polyester swatches in a solution of SRP (1.0% w/w), with swatches allowed to air-dry before sputter coating with a gold–palladium conducting layer. SEM images were taken at 500× magnification (Figure 4), and show morphological changes due to SRPs deposition on the surface. For the untreated surface (Nil), imaging showed the presence of an irregular textured surface with some sharp raised elements present on the surface of the fiber. After treating the fabrics with SRP, a noticeable change in surface morphology can be observed. For example, treatment with polymers **P1**, **P2** and **P7** appeared to smooth over the irregularities in the fiber, resulting in a more even surface. Additionally, polymer was observed to collect in the small gaps between overlapping fibers, as seen in images of surfaces treated these polymers (Figure 4; feature 1), potentially eliminating sites of soil deposition. Although this effect was also observed for surfaces treated with **P5** and **P6**, the polymer deposits on the fibers appeared overall more irregular than in the case of **P1**, **P2** and **P7**. Additional localized regions of a higher density deposit polymer were also seen, particularly for **P3** and **P4**, (Figure 4; feature 2), which the surface of the fiber similar in appearance to the polyester reference image, which was not treated with SRPs.

To explore potential differences in the affinity of SRPs for PET surfaces, we calculated surface binding energies for a selection of SRPs of varying surface activities. Here, we used a large series of energy minimization calculations to study the binding of the hydrophobic core to a model PET surface using an implicit solvent approximation (SI Sections 7.4, 7.8). As expected, the calculations demonstrated that the hydrophobic cores of all polymers engage in favorable noncovalent binding to the surface, although noticeably stronger binding is seen for the core of the terephthalate-2,5-pyridine dicarboxylate copolymer **P7** and for the reference SRP, **P8** (Table 2). Interestingly, very small differences in binding energies are seen between favorably performing **P1** and poorly performing **P5**, suggesting that the difference in the behavior of these two polymers may be a consequence of reduced deposition on the surface rather than a reduced affinity of the respective central hydrophobic block for the PET surface. We therefore explored the behavior of SRPs in solution, in order to probe the factors that may contribute to reduced deposition at the interface.

**Table 2:** Mean Binding Free Energies for Oligomer Cores with a PET Surface^a^

Oligomer core	Binding energy/kJ mol^–1^	Standard error/kJ mol^–1^	Standard deviation/kJ mol^–1^
**P1**	–62.3	0.8	34.9
**P5**	–61.4	0.8	35.3
**P7**	–70.9	0.9	40.9
**P8**	–69.1	0.9	38.5

a Values are given as an average of 1878 independent realizations of the minimization process, providing excellent sampling of the surface.

### Solution Self-Assembly

**Figure 5:**
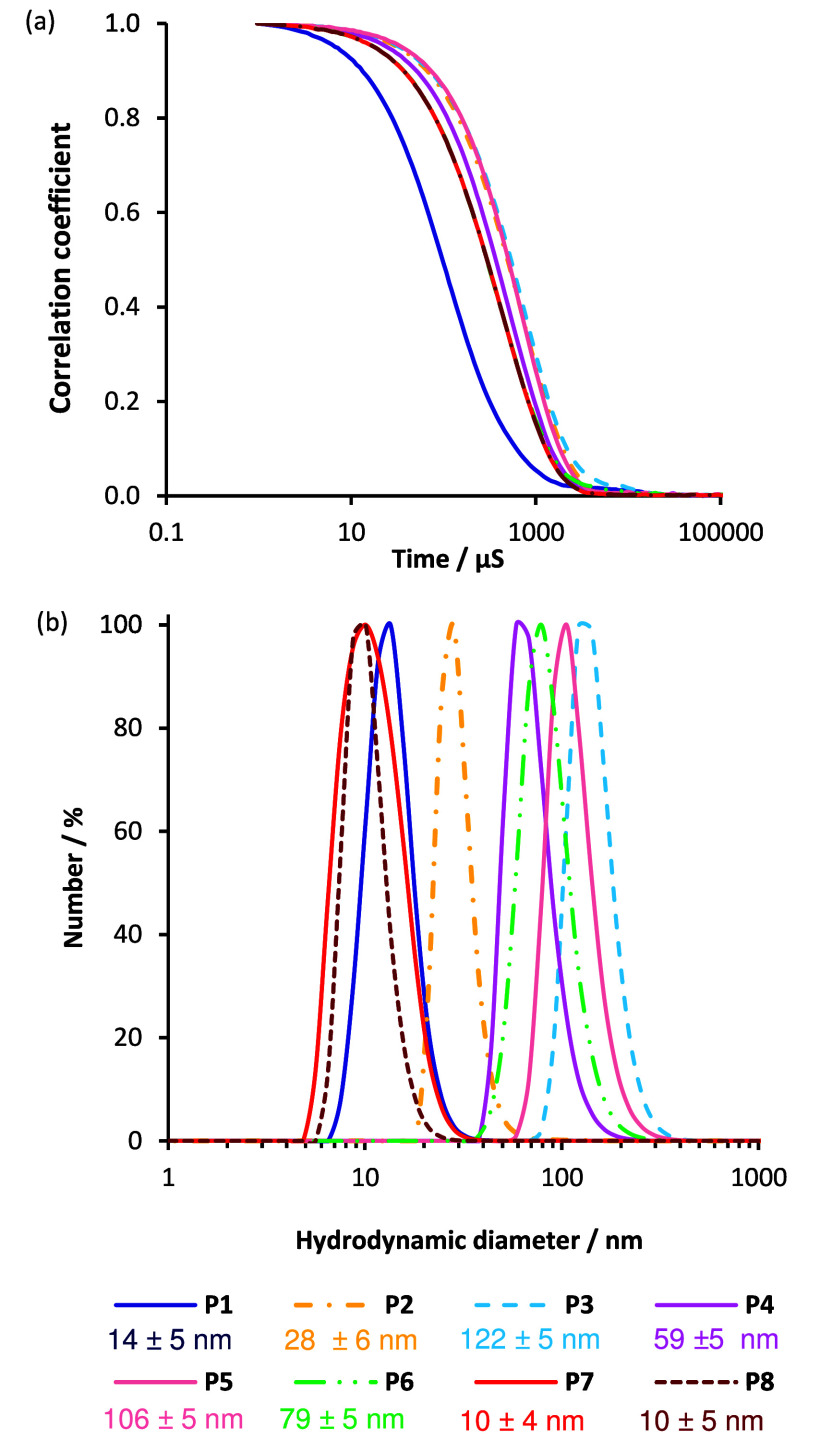
(a) DLS correlation functions and (b) normalized number-average particle size distributions with indicative number-average *D*_h_ for SRPs in an aqueous solution (1.0% w/w), with measurements recorded at 35 °C.

DLS analysis was performed on 1.0% w/w aqueous solutions of SRPs, with measurements recorded at 35 °C. In all cases, the presence of nanoscale aggregates was observed, with differences in diffusion rates evident from the correlation functions obtained (Figure 5a). In some cases, the intensity distributions were noted to be multimodal (SI Figure S4) and the CONTIN algorithm^33,34^ was used to deconvolute the signal and generate number average size distributions to allow for comparison of average aggregate sizes (Figure 5b; shown with indicative number average hydrodynamic diameters (*D*_h_)). Interestingly, we noted a correlation between apparent aggregate size and antiredeposition performance through comparison against the normalized ΔWI values observed (Figure 2b, Figure 5b). Notably, SRPs which form smaller aggregates displayed markedly better performance than those which form larger aggregates, despite their similar *M*_n_ values (Table 1). **P1**, for instance, was found to generate relatively small aggregates with an indicative *D*_h_ of 14 ± 5 nm, similar to those observed using the reference SRP, **P8** (10 ± 5 nm), which displays a similar molecular weight. Poorly performing polymers **P3** and **P5**, however, were shown to form larger aggregates with indicative *D*_h_ 122 ± 5 nm and 106 ± 5 nm, respectively, suggesting that these aggregates contain much higher numbers of polymeric species. This observation suggests that isomerism within the aromatic dicarboxylate component plays a significant role in the self-assembly of SRPs in solution, and that these differences can drastically affect soil-release performance when polymers are incorporated in detergent formulations. The presence of larger self-aggregates in aqueous solution is correlated with poor deposition of SRPs onto fabric surfaces, and may explain the observed differences in performance. **P7**, the copolymer demonstrated to display favorable performance, formed aggregates with indicative *D*_h_ 10 ± 4 nm, similar to that of **P8**, and consistent with the trend identified.

**Figure 6:**
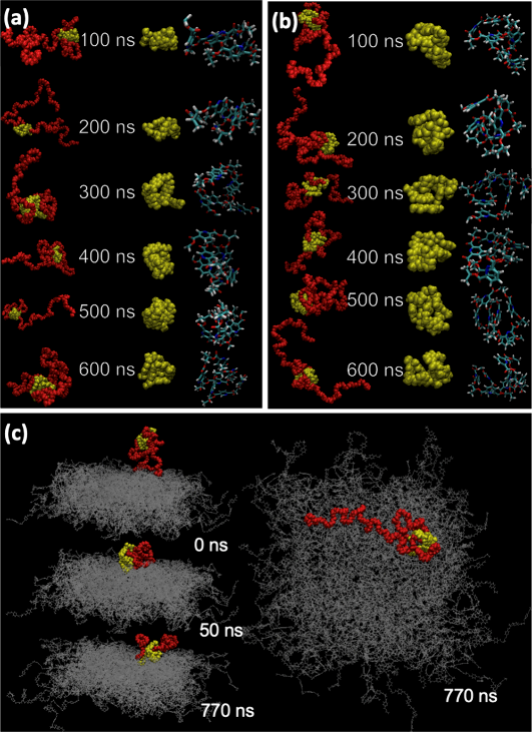
Time snapshots showing the folding and refolding of the hydrophobic core for (a) **P1** and (b) **P5** in water, demonstrating that the hydrophobic core of **P1** is more effectively shielded by the PEG corona than that of **P5**. (c) Capture of **P8** by a polyester surface at different simulation times after an initial 5 ns equilibration run.

The solution self-assembly behavior of SRPs was further investigated though molecular modeling. Atomistic molecular dynamics simulations for the SRPs in solution show that the hydrophobic parts of the molecule self-organize into folded structures facilitated by π- π stacking, as typically seen in aqueous solutions of chromonic liquid crystals.^35,36^ Here, the self-assembly of aromatic rings partially shields the hydrophobic parts of the polymer from interactions with water and the PEG chains form a corona around the central aromatic core (Figure 6a-b, SI Section 7.5). The folding behavior of some of the hydrophobic cores, dependent on structural isomerism within the pyridine dicarboxylate unit, was observed to be markedly different to others, leading to the PEG chains having different abilities to shield the cores from interactions with water molecules in the different polymers. This effect can be quantified by the number of hydrophobic core–water interactions within a defined cutoff (SI Table S1). The larger numbers of core-water interactions for 2,6-pyridine dicarboxylate derived **P5** (in comparison to 2,5-pyridine dicarboxylate derived **P1** and the reference SRP **P8**) are indicative of a poorly shielded hydrophobic core that leads to the formation of large aggregates in solution (SI Section 7.10).

Comparison of the behavior of polymers **P1** and **P5** simulated in a 2% w/w solution (SI Section 7.6.2) show the stronger driving force for aggregation in **P5** leads to the formation of large aggregates, while, for **P1**, monomers and small aggregates remain after 400 ns of simulation. Interestingly, if simulations are carried out on an analogue of **P1** with shorter PEG chains (mPEG-500; **P9**), the shielding of the core–water interactions becomes sufficiently poor for aggregation to occur extremely rapidly (within 125 ns for a 2% w/w solution) leading to large aggregates where the PEG chains are unable to shield the core from water (SI Figure S14). To verify this behavior, we synthesized **P9** and subjected it to DLS analysis, verifying that larger aggregates are formed when polymers display shorter PEG blocks (SI Figure S5), with indicative number-average *D*_h_ 54 ± 4 nm. **P9** was additionally evaluated for antiredeposition performance (SI Figure S3), and displayed no benefit, underlining the correlation between aggregate size and soil-release performance.

The differences in aggregate size appear to account for the observed differences in the anti-redeposition and soil release performance of **P1**-**P7**. Direct simulation of the capture of SRPs by a model PET surface (Figure 6c, SI Section 7.7) indicates that the adsorption of individual polymer chains is a spontaneous process, and even poorly performing polymers display significant affinity to PET (Table 2, **P5**). However, we hypothesize that surface adsorption of large aggregates (of the form shown in Figure S11 for **P5**), which are entirely surrounded by PEG chains is unlikely and therefore the formation of larger aggregates in aqueous solution is strongly correlated to poor performance.

## Conclusions

We have synthesized a series of SRPs using pyridine dicarboxylate monomers accessible from biomass, presenting a more sustainable route to the production of these key detergent additives which enable the efficient cleaning of textiles at lower wash cycles with reduced water consumption. SRPs containing 2,5-pyridine dicarboxylate units (**P1**-**2**, **P7**) were shown to match or exceed the performance of a reference SRP (**P8**) in anti-redeposition and soil-release performance tests, while SRPs containing 2,4- (**P3**-**4**) or 2,6-pyridine dicarboxylate units (**P5**-**6**) displayed markedly diminished performance. All polymers tested were observed to be capable of modifying PET surfaces and rendering them hydrophilic, in line with the expected mechanism of action of SRPs. Molecular modeling studies additionally support this mode of action, with calculated free energies of binding for the central hydrophobic block of both favorably performing and poorly performing SRPs suggesting that adsorption of the central hydrophobic block onto the PET surface is thermodynamically favorable. Interestingly, although larger free energies of binding are calculated for polymers that display the most favorable soil-release performance, differences in surface modification behavior for the series of SRPs tested appear to correlate more closely with their solution self- assembly behavior, rather than their interactions with the surface. Aggregation studies conducted using DLS suggest that favorably performing polymers form smaller aggregates in solution, with larger aggregate sizes associated with poor performance. Molecular modeling studies demonstrated differences in folding of the hydrophobic central block which affected the ability of the hydrophilic corona to effectively shield the core of the polymer, rationalizing the observed differences in aggregate size. We propose that within larger self-assembled aggregates, the hydrophobic central block of the SRP is buried within the core of the aggregate, shielded completely from the external environment by the PEG corona, and unable to access the PET surface in order to facilitate deposition.

This model provides molecular-level insight into the mechanism of action of SRPs in modifying the surfaces of textiles. These insights will guide the design of next-generation biosourced SRPs, with the observed correlations between solution self-assembly behavior, surface binding energies and performance offering possibilities to further improve the efficacy of SRPs through tuning steric interactions within the polymer chain, and the incorporation of substituents onto aromatic units within the hydrophobic core.

## Materials and Methods

### General Experimental Details

All reagents were purchased from Fisher, Merck or Fluorochem and used as received. All solvents were purchased from Fisher Scientific or Merck, and were HPLC grade. ^1^H NMR spectra were recorded on a Bruker Avance III spectrometer, with ^1^H at 400 MHz. Infrared spectra were recorded using a PerkinElmer Frontier FT-IR spectrometer, across a range of 4,000–500 cm^–1^. Polyester fabrics were purchased from WFK Testgewebe GmbH. A representative laundry formulation without soil-release polymers was provided by P&G (Newcastle Innovation Centre). For antiredeposition performance tests, polyester sheets loaded with BS2004 soil (SBL) were acquired from WFK Testgewebe GmbH and are composed of a synthetic soil mixture of vegetable oil, synthetic sebum, and solid particles such as kaolin and carbon black. Images of the polyester tracers were collected before and after washing with a Konica Minolta: CM-3630A reflection spectrophotometer. Images were analyzed using SpectraMagicNX software to determine the whiteness degree of fabrics. For the soil release performance test, dirty motor oil was acquired from Warwick Equest and a DigiEye reflection spectrophotometer was used to collect images that were then analyzed using DigiEye software.

### Polymer Synthesis and Characterization

Detailed synthetic procedures for the preparation of polymers **P1–P9**, and associated characterization data (^1^H NMR spectra, FT-IR spectra, gel permeation chromatography, DLS) may be found in the Supporting Information. Gel permeation chromatography measurements were conducted using an Agilent 1260 instrument equipped with differential refractive index detector, a variable wavelength UV–vis detector and a pair of PL aquagel–OH 8 μm Mixed-M columns (300 × 7.5 mm) with a guard column (Polymer Laboratories Inc.), connected in series. Chromatography was performed in 0.01 M NaNO_3(aq)_, (1.0 mL/min) at 35 °C. Near monodisperse PEO standards (Agilent) were used for calibration. Samples were prepared to a concentration of 5 mg/mL by dissolving 15 mg of SRP in 0.01 M NaNO_3__(aq)_. Samples were filtered using a sterile polyether sulfone syringe filter (0.2 μm). Hydrodynamic diameters (*D*_h_) of polymers in aqueous solutions (1.0% w/w) were determined by dynamic light scattering (DLS). The DLS instrumentation consisted of a Malvern Instruments Zetasizer operating at 35 °C with a 633 nm laser module. Measurements were made at a detection angle of 173° (back scattering), and Malvern Zetasizer software (version 8.02) was used to analyze the data. All determinations were made in triplicate. Samples were prepared by dissolving the SRP (100 mg) in 10 mL deionized water (1.0% w/w), the resulting solution was then filtered using a sterile polyether sulfone syringe filter (0.2 μm) into a 3 mL quartz cuvette.

### Contact Angle Measurements

Model surfaces for contact angle measurement were prepared by dissolving amorphous polyethylene terephthalate (amPET) in CHCl_3_ to give a 1% w/w solution, which was then spin-coated onto an acetone-cleaned silicon wafer at 2000 rpm for 30 s. These PET surfaces were then modified with SRP by leaving the PET silicon wafer to soak in a 1% w/w SRP solution (30 mg, 3 mL) for 40 min, and left to dry upside down to allow excess SRP to run off the surface. A 5 μL droplet of deionized water was then placed on each of the treated surfaces and the contact angle was measured at room temperature. The images taken were imported and processed using ImageJ 1.54g software using the drop snake plugin to calculate the left and right contact angles of the droplet. The reference surfaces of an unmodified PET surface and one with just methoxy polyethylene glycol used to allow for a direct comparison to investigate the surface capabilities of the SRP-modified surface.

### Scanning Electron Microscopy

Scanning electron microscope images were then obtained using a Carl Zeiss 300VP electron microscope operated at 5 kV, 300 μm aperture. Samples for image analysis were prepared by soaking 1 × 1 cm^2^ polyester swatches in a 1.0% w/w solution of SRP (30 mg in 3 mL deionized water), with swatches allowed to air-dry before sputter coating with a gold–palladium conducting layer of around 38 nm, using a Cressington sputter 108 autocoater.

## Supporting Information Available

Synthetic procedures and characterization data, experimental details of performance test, molecular modeling studies (PDF)
