# Supplementary material for: Surface Modification of Polyesters Using Biosourced Soil-Release Polymers
Source: JACS Au. 2025 Feb 5;5(2):666–74. doi: 10.1021/jacsau.4c00908 (PMC11862958; doi:10.1021/jacsau.4c00908)
Supplement: Supplementary file 1 — au4c00908_si_001.pdf [file au4c00908_si_001.pdf]

# Surface modification of polyesters using biosourced soil-release polymers

Matthieu Starck,[a] Emanuella F. Fiandra,[a] Josephine Binks,[a] Gang Si,[b] Ruth Chilton,[b] Mark Sivik,[c] Richard L. Thompson,[a] Jing Li, [a] Mark R. Wilson[a] and Clare S. Mahon[a,*]

[a] Department of Chemistry, Durham University, Durham, DH1 3LE, [b] The Procter & Gamble Newcastle Innovation Centre, Whitley Rd, Newcastle upon Tyne, NE12 9BZ United Kingdom, [c] Procter & Gamble Company, Fabric & Home Care Innovation Centre, Cincinnati, Ohio, 45217, United States

***1. General experimental details*** ***2***

***2. Synthesis and characterisation*** ***2***
2.1 General procedure for synthesis of **P1-P6** 2
2.2 Synthesis of **P7** 2
2.3 Synthesis of **P8** 3
2.4 Synthesis of **P9** 3
2.5 Gel permeation chromatography 3
2.6 FT-IR spectroscopy 5

***3. Performance testing*** ***6***
3.1 Anti-redeposition performance test 6
3.2 Soil-release performance test 6

***4. Contact angle measurements*** ***7***

***5. Scanning Electron Microscopy (SEM)*** ***7***

***6. Dynamic light scattering*** ***7***

***7. Molecular modeling*** ***10***
7.1 Generation of force fields for molecular dynamics and energy minimisation work 10
7.2 Molecular dynamics (MD) calculations 10
7.3 Generation of a PET surface 10
7.4 Energy minimisation of oligomer cores at a PET surface 11
7.5 Single polymer MD in water 12
7.6 MD for ten polymer molecules in a water 14
7.7 Spontaneous adsorption of a reference SRP onto a PET surface 15
7.8 Binding energies 17
7.9 Quantum chemical calculations of torsional energy barriers 17
7.10 Effects of isomerism within pyridine dicarboxylate unit on aggregation 20

***8. NMR spectra*** ***21***

## 1. General experimental details

All reagents were purchased from Fisher, Merck or Fluorochem and used as received. All solvents were purchased from Fisher Scientific or Merck, and were HPLC grade. $^1$H NMR spectra were recorded on a Bruker Avance III spectrometer, with $^1$H at 400 MHz. Infrared spectra were recorded using a Perkin Elmer Frontier FT-IR spectrometer, across a range of 4,000 - 500 cm$^{-1}$. Polyester fabrics were purchased from WFK Testgewebe GmbH. A representative laundry formulation without soil-release polymers was provided by P&G (Newcastle Innovation Centre). For the anti-redeposition performance test, polyester sheets loaded with BS2004 soil (SBL) were acquired from WFK Testgewebe GmbH and are composed of a synthetic soil mixture of vegetable oil, synthetic sebum, and solid particles such as kaolin and carbon black. Images of the polyester tracers were collected before and after washing with a Konica Minolta: CM-3630A reflection spectrophotometer. Images were analysed using SpectraMagicNX software to determine the whiteness degree of fabrics. For the soil release performance test, dirty motor oil was acquired from Warwick Equest and a DigiEye reflection spectrophotometer was used to collect images that were then analysed using DigiEye software.

## 2. Synthesis and characterisation

### 2.1 General procedure for synthesis of P1-P6

In reactor fitted with an overhead stirrer, a distillation bridge, and an argon inlet with in-line bubbler, 2,5-pyridinedicarboxylic acid dimethyl ester **1** (2.90 g, 15.0 mmol) was combined with 1,2-propanediol **5** (46.0 g, 600 mmol), mPEG2000 **6** (6.0 g, 3.0 mmol), sodium acetate (0.2 g, 2.5 mmol) and tetraisopropyl orthotitanate (0.8 mL, 3.0 mmol). The reaction mixture was heated at 170 °C under an argon atmosphere with constant stirring for 3 h, before the temperature was increased to 210 °C for an additional 1 h. The pressure within the vessel was decreased gradually to 1 mbar over 30 minutes, and the reaction mixture was heated to 210 °C, under reduced pressure with stirring for 3 h. The reaction mixture was then cooled to room temperature, and the crude polymer dissolved in tetrahydrofuran (150 mL) with sonication. The solution was centrifuged (3845 *g*, 5 min) and the supernatant filtered. The filtrate was evaporated, yielding a brown oil which was dissolved in tetrahydrofuran (20 mL). Diethyl ether (250 mL) was added, affording a brown precipitate which was isolated by filtration and washed with cold diethyl ether and dried under high vacuum (**P1**, 5.7 g, 70% yield).

**P1:** $^1$H NMR (298 K, 400 MHz, $CDCl_3$) $\delta_H$ 9.29 (br s, 7 H, C*H* pyridine), 8.41 (br s, 7 H, C*H* pyridine), 8.17 (br s, 7 H, C*H* pyridine), 5.69 – 5.33 (2 m, 6 H, C*H* 1,2-propanediol), 4.70 – 4.20 (2 m, 12 H, C$H_2$ 1,2-propanediol), 3.65 (br s, 350 H, C$H_2$ PEG), 3.39 (s, 6 H, C$H_3$O-PEG), 1.60-1.00 (m, 18 H, C$H_3$ 1,2-propanediol).

**P2:** $^1$H NMR (298 K, 400 MHz, $CDCl_3$) $\delta_H$ 9.28 (br s, 11H, C*H* pyridine), 8.41 (br s, 11H, C*H* pyridine), 8.16 (br s, 11H, C*H* pyridine), 5.70 – 5.30 (2 m, 10 H, C*H* 1,2-propanediol), 4.70 – 4.20 (2 m, 20 H, C$H_2$ 1,2-propanediol), 3.65 (br s, 350 H, C$H_2$ PEG), 3.39 (s, 6H, C$H_3$O-PEG), 1.60-1.00 (m, 30 H, C$H_3$ 1,2-propanediol).

**P3:** $^1$H NMR (298 K, 400 MHz, $CDCl_3$) $\delta_H$ 8.90 (br s, 7 H, C*H* pyridine), 8.62 (br s, 7 H, C*H* pyridine), 8.00 (br s, 7 H, C*H* pyridine), 5.70 – 5.30 (m, 6 H, C*H* 1,2-propanediol), 4.70 – 4.20 (m, 12 H, C$H_2$ 1,2-propanediol), 3.66 (br s, 350 H, C$H_2$ PEG), 3.39 (s, 6 H, C$H_3$O-PEG), 1.60-1.00 (m, 18 H, C$H_3$ 1,2-propanediol).

**P4:** $^1$H NMR (298 K, 400 MHz, $CDCl_3$) $\delta_H$ 8.89 (br s, 11 H, C*H* pyridine), 8.60 (br s, 11 H, C*H* pyridine), 8.01 (br s, 11 H, C*H* pyridine), 5.70 – 5.30 (2 m, 10 H, C*H* 1,2-propanediol), 4.70 – 4.20 (2 m, 20 H, C$H_2$ 1,2-propanediol), 3.66 (br s, 350 H, C$H_2$ PEG), 3.39 (s, 6 H, C$H_3$O-PEG), 1.60-1.00 (m, 33 H, C$H_3$ 1,2-propanediol).

**P5:** $^1$H NMR (298 K, 400 MHz, $CDCl_3$) $\delta_H$ 8.28 (br s, 14 H, C*H* pyridine), 8.02 (br s, 7 H, C*H* pyridine), 5.70 – 5.30 (2 m, 6H, C*H* 1,2-propanediol), 4.70 – 4.20 (2 m, 12 H, C$H_2$ 1,2-propanediol), 3.65 (br s, 350 H, C$H_2$ PEG), 3.39 (s, 6H, C$H_3$O-PEG), 1.60-1.00 (m, 18 H, C$H_3$ 1,2-propanediol).

**P6:** $^1$H NMR (298 K, 400 MHz, $CDCl_3$) $\delta_H$ 8.24 (br s, 22 H, C*H* pyridine), 8.02 (br s, 11 H, C*H* pyridine), 5.70 – 5.30 (2 m, 10 H, C*H* 1,2-propanediol), 4.70 – 4.20 (2 m, 20 H, C$H_2$ 1,2-propanediol), 3.66 (br s, 350 H, C$H_2$ PEG), 3.39 (s, 6H, C$H_3$O-PEG), 1.60-1.00 (m, 30 H, C$H_3$ 1,2-propanediol).

### 2.2 Synthesis of P7

In a reactor fitted with an overhead stirrer, a distillation bridge, and an argon inlet with in-line bubbler, 2,5-pyridinedicarboxylic acid dimethyl ester **1** (1.2 g, 6.0 mmol) was combined with dimethyl terephthalate (3.0

g, 15.0 mmol), 1,2-propanediol **5** (46.0 g, 600 mmol), mPEG2000 6 (6.0 g, 3.0 mmol), sodium acetate (0.2 g, 2.5 mmol) and tetraisopropyl orthotitanate (0.8 mL, 3.0 mmol). The reaction mixture was heated at 170°C under an argon atmosphere with constant stirring for 3 h, before the temperature was increased to 210 °C for an additional 1 h. The pressure within the vessel was decreased gradually to 1 mbar over 30 minutes, and the reaction mixture was heated to 210 °C under reduced pressure with stirring for 3 h. The reaction mixture was then cooled to room temperature, and crude polymer dissolved in tetrahydrofuran (150 mL) with sonication. The solution was centrifuged (3845 *g*, 5 min) and the supernatant filtered. The filtrate was evaporated, yielding an oil which was dissolved in tetrahydrofuran (20 mL). Diethyl ether (250 mL) was added, affording a brown precipitate which was isolated by filtration and washed with cold diethyl ether and then further dried (**P7**, 7.0 g, 78% yield).

$^1$H NMR (298 K, 400 MHz, $CDCl_3$) $\delta_H$ 9.29 – 8.39 (3 br s, 9 H, C*H* pyridine), 8.06 (br s, 30 H, C*H* terephthalate), 5.70 – 5.15 (2 m, 9 H, C*H* 1,2-propanediol), 4.70 – 4.10 (2 m, 18 H, $CH_2$ 1,2-propanediol), 3.65 (br s, 350 H, $CH_2$ PEG), 3.39 (s, 6 H, $CH_3$O-PEG), 1.55-1.00 (m, 27 H, $CH_3$ 1,2-propanediol).

### 2.3 Synthesis of P8

In a reactor fitted with an overhead stirrer, a distillation bridge, and an argon inlet with in-line bubbler, dimethyl terephthalate **4** (3.0 g, 15.0 mmol) was combined with 1,2-propanediol **5** (46.0 g, 600 mmol), mPEG2000 **6** (6.0 g, 3.0 mmol), sodium acetate (0.2 g, 2.5 mmol) and tetraisopropyl orthotitanate (0.8 g, 2.8 mmol). The reaction mixture was heated at 170 °C under an argon atmosphere with constant stirring for 3 h, before the temperature was increased to 210 °C for an additional 1 h. The pressure within the vessel was decreased gradually to 1 mbar over 30 minutes, and the reaction mixture was heated to 210 °C under reduced pressure with stirring for 3 h. The reaction mixture was then cooled to room temperature, and crude polymer dissolved in tetrahydrofuran (150 mL) with sonication. The solution was centrifuged (3845 *g*, 5 min) and the supernatant filtered. The filtrate was evaporated, yielding an oil which was dissolved in tetrahydrofuran (20 mL). Diethyl ether (250 mL) was added, affording a white solid which was isolated by filtration and washed with cold diethyl ether and dried under high vacuum (**P8**, 6.8 g, 87% yield).

$^1$H NMR (298 K, 400 MHz, $CDCl_3$) $\delta_H$ 8.08 (br s, 24 H, C*H* terephthalate), 5.57 (s, 5 H, C*H* 1,2-propanediol), 4.60 – 4.48 (m, 10 H, $CH_2$ 1,2-propanediol), 3.65 (br s, 380 H, $CH_2$ PEG), 3.39 (s, 6 H, $CH_3$O-PEG), 1.53-1.07 (m, 15 H, $CH_3$ 1,2-propanediol).

### 2.4 Synthesis of P9

In reactor fitted with an overhead stirrer, a distillation bridge, and an argon inlet with in-line bubbler, 2,5-pyridinedicarboxylic acid dimethyl ester **1** (4.5 g, 23 mmol) was combined with 1,2-propanediol **5** (18.0 g, 240 mmol), mPEG500 (2.3 g, 4.6 mmol), sodium acetate (0.2 g, 2,5 mmol) and tetraisopropyl orthotitanate (1.2 g, 4.6 mmol). The reaction mixture was heated at 170 °C under an argon atmosphere with constant stirring for 3 h, before the temperature was increased to 210 °C for an additional 1 h. The pressure within the vessel was decreased gradually to 1 mbar over 30 minutes, and the reaction mixture was heated to 210 °C under reduced pressure with stirring for 3 h. The reaction mixture was then cooled to room temperature, and the crude polymer dissolved in tetrahydrofuran (100 mL) with sonication. The solution was centrifuged (3845 *g*, 5 min) and the supernatant filtered. The filtrate was evaporated, yielding a brown oil. Diethyl ether (250 mL) was added, with no precipitate formed, and oil was decanted and dried to yield P9 as dark red oil which was dried under high vacuum (**P9**, 3.67 g, 81% yield).

$^1$H NMR (298 K, 400 MHz, $CDCl_3$) $\delta_H$ 9.34 (br s, 5 H, C*H* pyridine), 8.48 (br s, 5 H, C*H* pyridine), 8.23 (br s, 5 H, C*H* pyridine), 5.70 – 5.34 (m, 4 H, C*H* 1,2-propanediol), 4.69 – 4.28 (m, 8 H, $CH_2$ 1,2-propanediol), 3.67 (br s, 88 H, $CH_2$ PEG), 3.40 (s, 6 H, $CH_3$O-PEG), 1.54-1.33 (m, 18 H, $CH_3$ 1,2-propanediol).

### 2.5 Gel permeation chromatography

Gel permeation chromatography measurements were conducted using an Agilent 1260 instrument equipped with differential refractive index detector, a variable wavelength UV-Vis detector and a pair of PL aquagel-OH 8μm Mixed-M columns (300 x 7.5 mm) with a guard column (Polymer Laboratories Inc.), connected in series. Chromatography was performed in 0.01 M $NaNO_{3(aq)}$, (1.0 mL/min) at 35 °C. Near monodisperse PEO standards (Agilent) were used for calibration. Samples for gel permeation chromatography (GPC) were prepared to a concentration of 5 mg/mL by dissolving 15 mg of SRP (**P1**-**P8**) in 0.01 M $NaNO_3$., Samples were filtered using a sterile polyether sulfone syringe filter (0.2 μm).

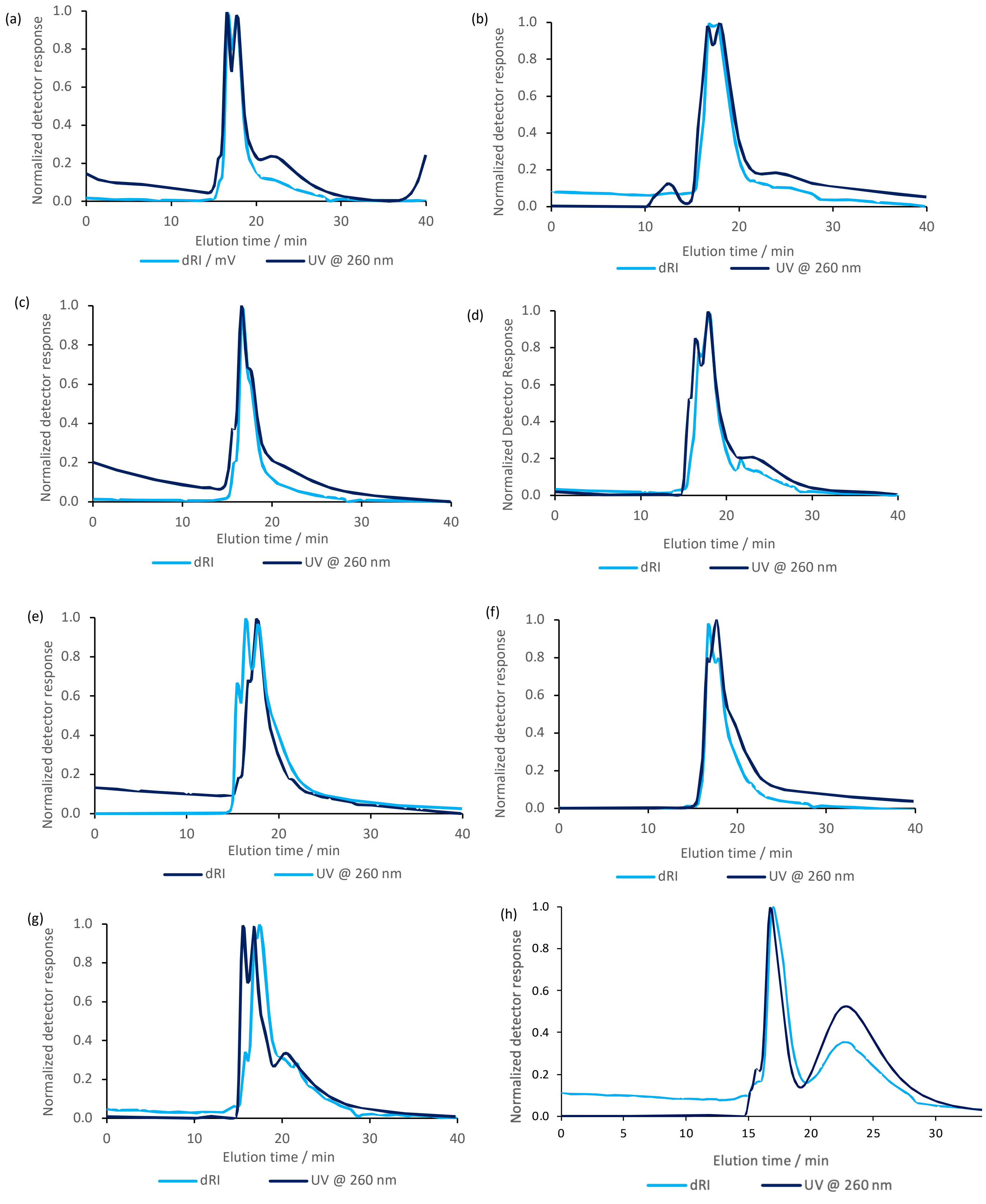


**Figure S1** Gel permeation chromatograms for **P1-P8** (a-h), performed using 0.01 M $NaNO_{3(aq)}$ (1.0 mL $min^{-1}$) at 35 °C. Molecular weights reported are determined through calibration against near monodisperse PEO standards.

## 2.6 FT-IR spectroscopy

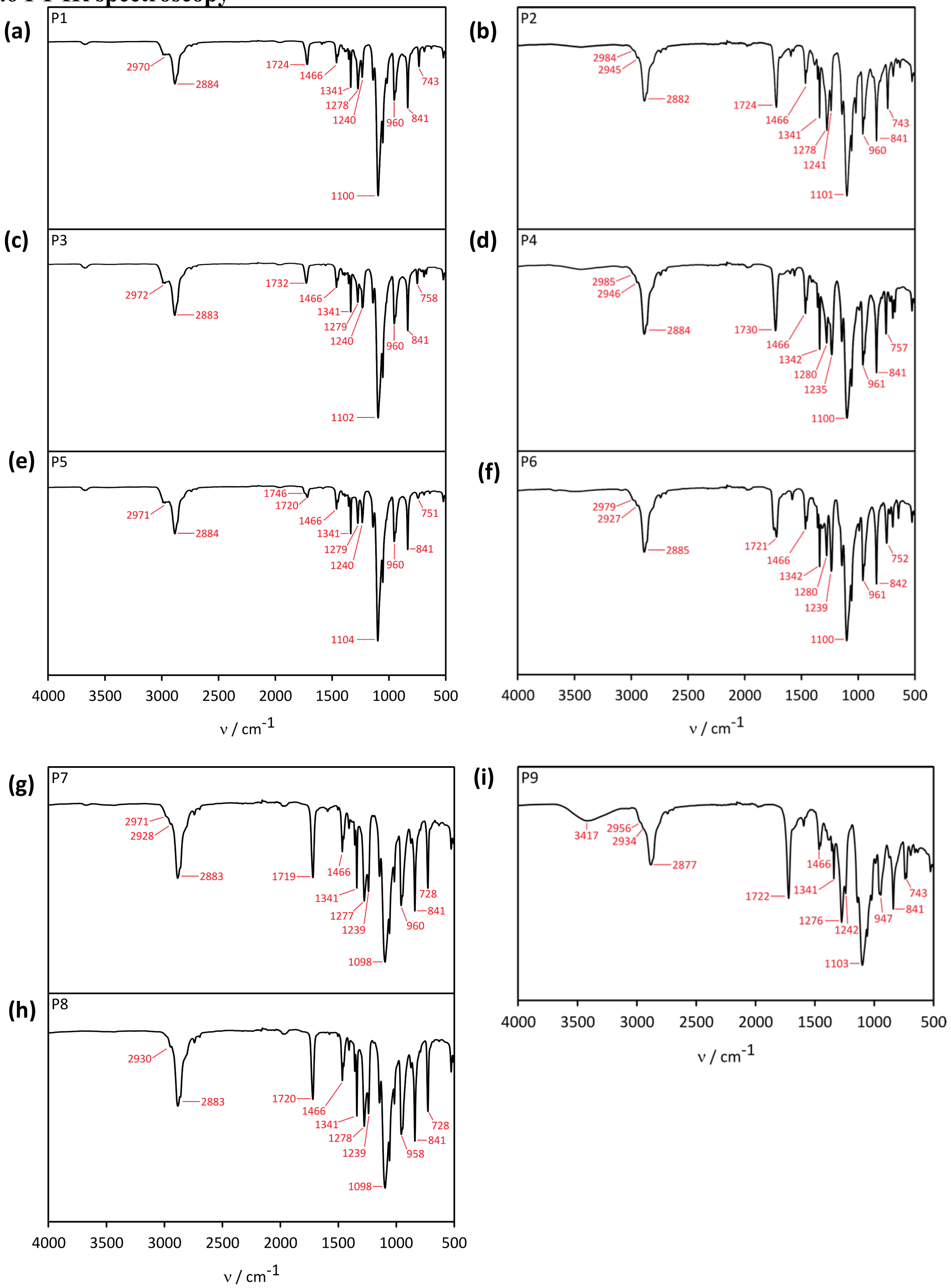


**Figure S2** FT-IR spectra of (a)-(i) **P1**-**P9**. Notable features include: aromatic C-H bond stretching at 2980 - 2930 $cm^{-1}$; PEG and propylene glycol C-H bond stretching are observed with an absorption at 2880 $cm^{-1}$; C=O bond stretching at 1720 - 1730 $cm^{-1,}$ with the absorption band 1280 $cm^{-1}$ corresponding to the C-O bonds stretching, characteristic of ester bonds. At 1466 $cm^{-1}$ and 1340 $cm^{-1}$, absorption bands correspond to the deformation of C-H bonds of the PEG and propylene glycol alkyl chain. The intense band at 1100 $cm^{-1}$ is characteristic of the C-O stretch of PEG ether groups. The bands at 960 and 840 $cm^{-1}$ correspond to the deformation 'out of plane' (oop) of C-H bonds of the aliphatic groups and the band at 729 to 758 $cm^{-1}$ corresponds to the deformation 'oop' of the aromatic C-H bonds.

## 3. Performance testing

### 3.1 Anti-redeposition performance test

Whiteness tests were performed in an automatic tergotometer system. Each wash load included four tracers of each type of fabric (polyester, knitted cotton, polycotton and polyspandex) that were imaged (Konica Minolta: CM-3630A); SBL swatches to simulate soil levels of a typical consumer, and knitted cotton and polycotton garments to reproduce the washing conditions of a consumer (60 g total fabric load, each fabric 5 x 5 $cm^2$). Samples were prepared by adding 1 wt.% SRP to a representative laundry formulation, which was then left to stand at room temperature for 18 h. Each wash load dose was diluted in hard water (21 gpg) in the tergotometer chambers to 50 ppm and mixed at 300 rpm for 1 min. Fabrics were then added to the chambers and mixed with an agitation speed of 300 rpm at 35 °C for 40 min, followed by two 5 minute 15 °C rinse cycles with an agitation speed of 208 rpm. Fabrics were collected from each chamber and exhausted SBL swatches were replaced with fresh ones. The washing cycle was performed again under the same conditions reported and were repeated four times in total. Tracers were then recovered at the end of the washing process and dried before undergoing image analysis to obtain the post reads (Konica Minolta: CM-3630A).

In addition to the data presented in the manuscript, we have evaluated the anti-redeposition performance of **P9**, the mPEG500 analogue of **P1**, and mPEG5000 i.e. an analogue of **P1**-**P8** without the hydrophobic central block. In each case polymers displayed no demonstrable benefit (Figure S3).

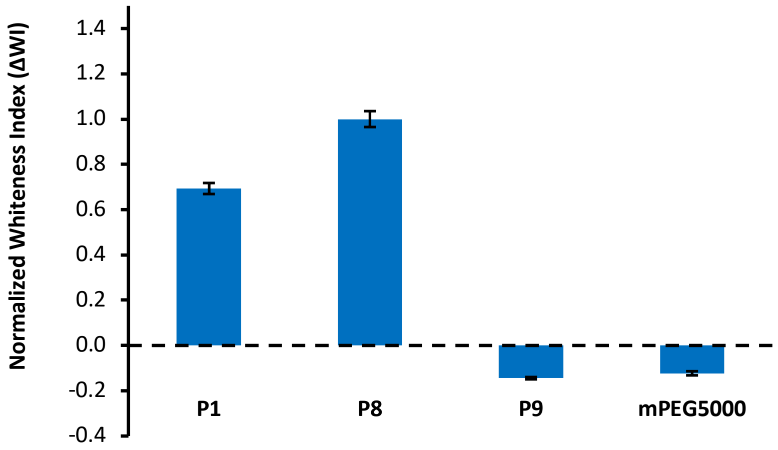


**Figure S3** Difference in whiteness index variation (ΔWI) of polyester tracers washed with a laundry detergent formulation with 1 % (w/w) SRP. The baseline 0.0 indicates the performance of the SRP-free negative control (Nil).

### 3.2 Soil-release performance test

5 x 5 $cm^2$ polyester tracers were pre-conditioned with SRP using an automatic tergotometer system. 1 % w/w aqueous SRP solutions were made up and left to stand at room temperature for 18 h. Samples were prepared by adding 1 % w/w SRP solution diluted in 1L city water (~8 gpg) in the tergotometer chambers to 50 ppm and mixed at 417 rpm for 1 minute. Four polyester tracers and knitted cotton (60 g total fabric load) were then added to each pot and mixed with an agitation speed of 300 rpm at 30 °C for 40 minutes to simulate the washing cycle, followed by two 5 minute 15 °C rinse cycles with an agitation speed of 208 rpm. Tracers were then recovered and dried. Dirty motor oil (200 µL) was then pipetted on each polyester tracer and allowed to wick overnight before performing image analysis (DigiEye).

A stain removal cycle was then performed to assess the effect of the SRP modified surfaces on DMO stain removal using the automatic tergotometer system. Each treatment was this time washed with a laundry detergent formulation containing no SRP, this allows assessment of the effects of pre-treating the fabric surface with SRP on stain removal in the subsequent wash phase. Wash conditions were the same as those used in the preconditioning. Tracers were then recovered at the end of the washing process and dried before undergoing image analysis to obtain the post reads (DigiEye).

## 4. Contact angle measurements

Model surfaces were prepared by dissolving amorphous polyethylene terephthalate (amPET) in $CHCl_3$ to give a 1 wt.% solution, which was then spin-coated onto an acetone-cleaned silicon wafer at 2000 rpm for 30 seconds. These PET surfaces were then modified with SRP by leaving the PET silicon wafer to soak in a 1% w/w SRP solution (30 mg, 3 mL) for 40 minutes, and left to dry upside down to allow excess SRP to run off the surface. A 5 µL droplet of deionised water was then placed on each of the treated surfaces and the contact angle was measured at room temperature. The images taken were imported and processed using ImageJ 1.54g software using the drop snake plugin to calculate the left and right contact angles of the droplet. The reference surfaces of an unmodified PET surface and one with just methoxy polyethylene glycol used to allow for a direct comparison to investigate the surface capabilities of the SRP-modified surface.

## 5. Scanning Electron Microscopy (SEM)

Samples for image analysis were prepared by soaking 1 x 1 $cm^2$ polyester swatches in a 1.0 % w/w solution of SRP (30 mg in 3 mL deionised water), with swatches allowed to air dry before sputter coating with a gold-palladium conducting layer of around 38 nm, using a Cressington sputter 108 autocoater. Scanning electron microscope images were then obtained using a Carl Zeiss 300VP Electron Microscope operated at 5 kV, 300 µm aperture.

## 6. Dynamic light scattering

Hydrodynamic diameters ($D_h$) of polymers in aqueous solutions (1.0% w/w) were determined by dynamic light scattering (DLS). The DLS instrumentation consisted of a Malvern Instruments Zetasizer operating at 35 ℃ with a 633 nm laser module. Measurements were made at a detection angle of 173° (back scattering), and Malvern Zetasizer software (version 8.02) was used to analyse the data. All determinations were made in triplicate. Samples were prepared by dissolving the SRP (100 mg) in 10 mL deionised water (1.0% w/w), the resulting solution was then filtered using a sterile polyether sulfone syringe filter (0.2 µm) into a 3 mL quartz cuvette.

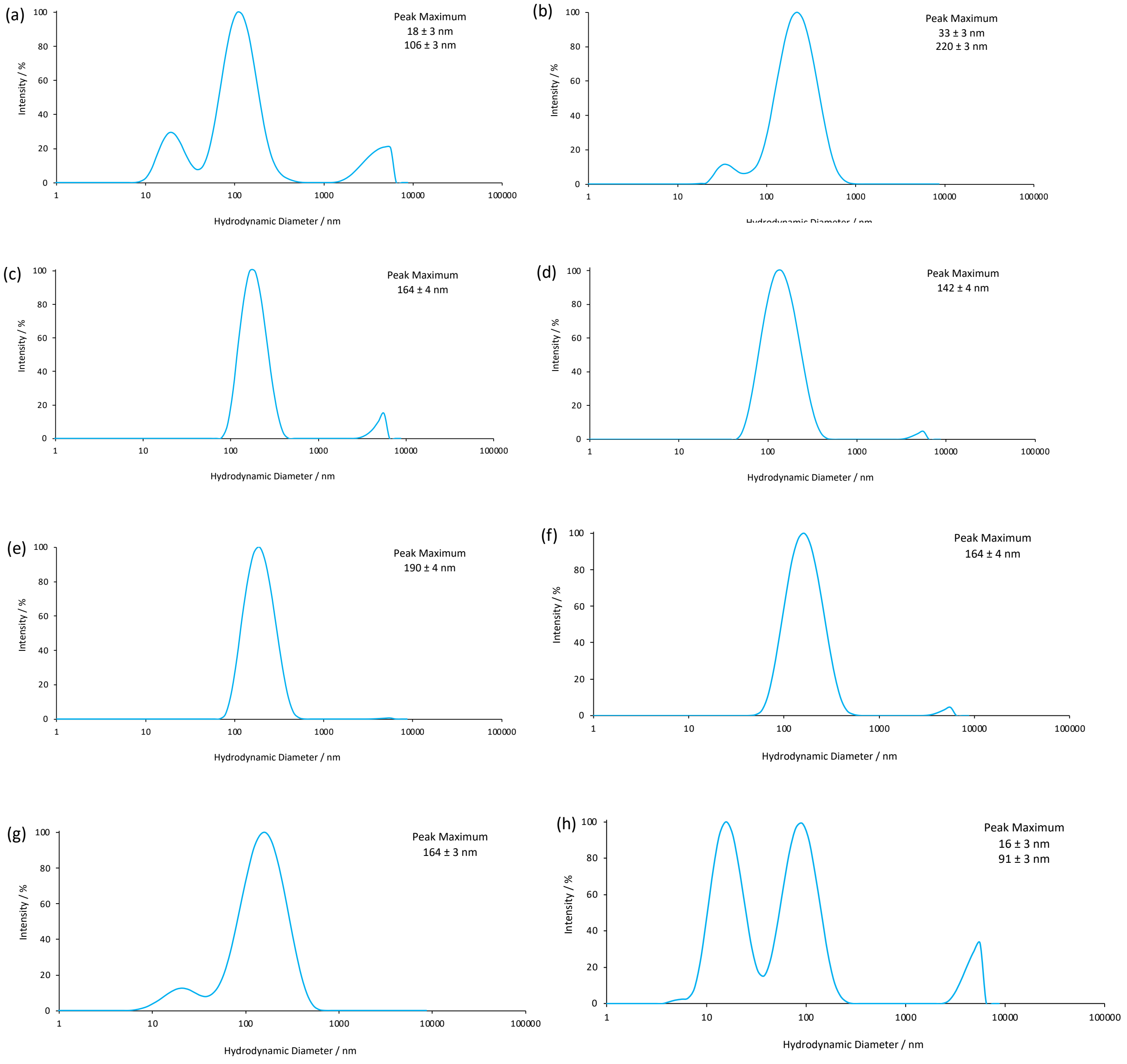


**Figure S4** The average size distribution by intensity for **P1**-**P8** (a)-(h) in aqueous solution (1.0% w/w) at 35 °C.

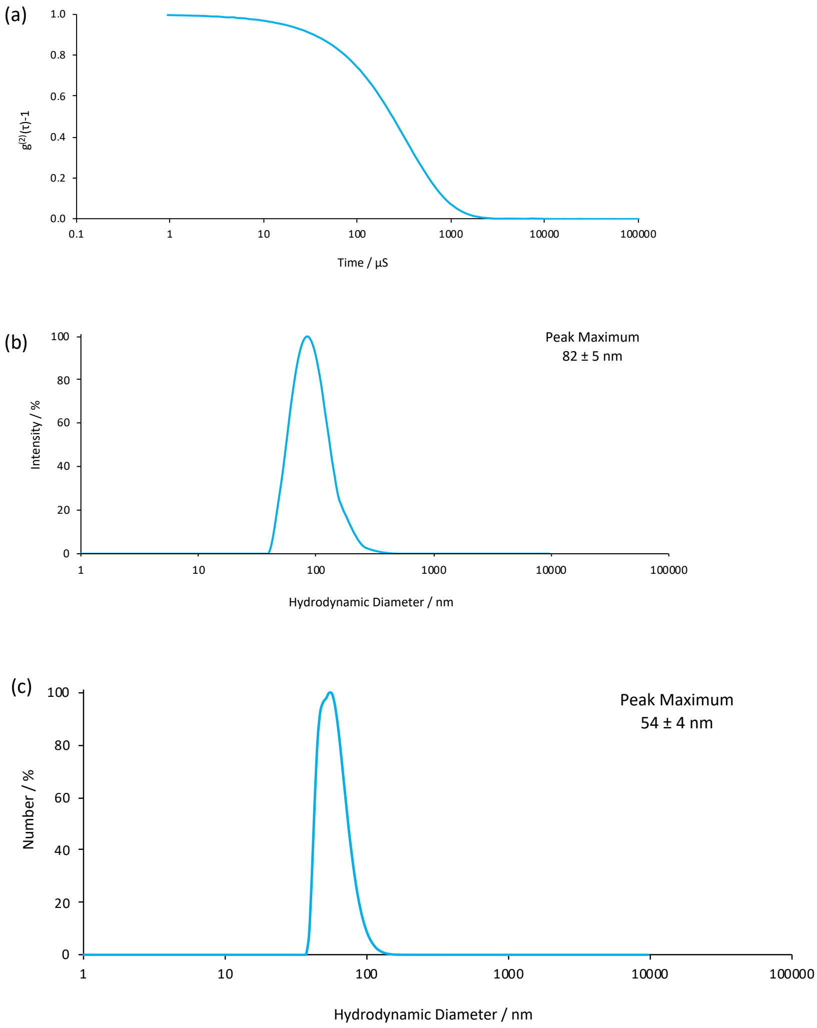


**Figure S5 (a)** DLS correlation function and normalised average particle size distribution by intensity **(b)** and number **(c)** for **P9** in an aqueous solution (1.0% w/w) at 35 °C.

## 7. Molecular modeling

### 7.1 Generation of force fields for molecular dynamics and energy minimisation work

Force fields for individual molecules were generated using AmberTools, version 23,[1] and employed AM1-BCC charges,[2,3] with the exception of the force field for PEG chains (discussed below). Energy minimisation and molecular dynamics calculations used the GAFF force field[4] updated to use enhanced torsional potentials fitted to energies from MP2/6-31+g(d,p) electronic structure calculations (ESI §7.9), following the methodology of Boyd and Wilson[5]. This procedure took into account the other elements of the force field in fitting the dihedrals, including the contributions from 1,4 non-bonded and 1,4 electrostatic interactions. The MP2 calculations were carried out using Gaussian 16.[6] The updated torsional parameters ensured a good representation of folding within the hydrophobic polymer cores.

For poly(ethylene glycol) ethers (glymes) the standard GAFF force field has been shown to be inaccurate when used to simulate glymes, overestimating both the density and dielectric constant.[7] However, an improved GAFF force fields for glymes was recently produced by Barbosa *et al*[7], the new force field with updated Lennard—Jones parameters and partial charges gives excellent agreement with experimental liquid phase densities and dielectric constants over a wide temperature range in the bulk phase. However, tests of the new force field for PEG chains in water[8] suggest that although this force field performs well, it performs slightly less well than the all-atom modified TraPPE-UA model of Fischer, J. *et al*[9] and predicts a slightly larger radius of gyration than seen in the light scattering experiments of Kawaguchi *et al*[10], i.e. effectively water is slightly too good a solvent for these chains. Recently, Sherck *et al*[11] have measured the end-to-end distance probability distributions of dilute poly(ethylene oxide) chains in aqueous solution using double electron−electron resonance (DEER) spectroscopy and shown that excellent agreement can be obtained between these data and molecular dynamics simulations. In this work the Lennard-Jones parameters of Barbosa *et al.* were used with GAFF2 and they investigated a series of RESP charge models. The charges used were slightly reduced from those used in references.[7, 8] In the current work, for compatibility with the hydrophobic cores, we have used the GAFF force field, but used the modified Lennard-Jones parameters of Barbosa *et al.,*[7] and RESP charges averaged over a series of glymes, which are very close to those used by Sherck et al.[11] For PEG45 chains these yield a value of $R_g$ of 14.3 Å, close to the values from Kawaguchi *et al*[10] 13.1 Å.

### 7.2 Molecular dynamics (MD) calculations

MD calculations were carried out using the Amber program.[12] Here, we studied single polymer molecules, built using the tleap program of AmberTools, energy minimised with the sander program of AmberTools, and then solvated with TIP3P water.

For these and all subsequent MD simulations, we initially energy minimised the system, warmed the system to 298.15 K over 20 ps, pre-equilibrated at constant-*NVT* for 20 ps and at constant-*NpT* for 20 ps to test stability, and finally ran an equilibration run at constant-*NpT* over 5 ns. For these simulations, we use a Lennard-Jones cutoff of 1.2 nm, model long-range electrostatics through the Particle Mesh Ewald method, use SHAKE constraints for bonds involving hydrogen atoms, use a 2 fs time step, a Langevin thermostat with collision frequency = 2 $ps^{-1}$, and for constant pressure runs we use the Amber Monte Carlo barostat. For the polymer configurations in water, MD production runs were typically carried out for at least 650 ns, with time-averaged measurements occurring over the final 600 ns.

For studies with a model PET surface, the surface was initially built (as described below in §7.3), then final configurations from the polymer simulations in water were combined into a simulation box with the model surface. This system was re-solvated, energy minimised, equilibrated and used for 770 ns production MD runs to study the adsorption of full polymers at a PET surface.

We also investigated the aggregation of 10 **P1** and **P5** molecules in water at two different concentrations (see below, §7.5-7.6), and we studied the folding of different oligomer cores in water using 100 ns production runs to provide input for the energy minimisation calculations (described below, §7.4).

### 7.3 Generation of a PET surface

To generate a suitable PET surface, we initially carried out simulations of 10mer molecules of PET in the liquid phase. Here we started from a gas of 200 10mers in a cubic box, compressed at 100 bar and 550 K to give an oligomer liquid melt, equilibrated at 550 K and 1 bar of pressure, and then cooled to 298.15 K. We then heated to 550 K and applied an anisotropic barostat to produce a thin polymer slab of approximate dimensions 10.8 x 10.8 x 6 nm. We then added free space above and below the slab and equilibrated at 550 K before slowly cooling to 298.15 K (below the glass transition).

### 7.4 Energy minimisation of oligomer cores at a PET surface

The model PET surface studied varies in surface roughness at the Angstrom level. Hence, to provide a representative model of the strength of polymer binding at the surface, we developed a protocol to sample a set of representative surface states (Figure S8). For these calculations, we used just the hydrophobic oligomer core, terminated at each end with $CH_2CH_2OH$ groups. We used independent (folded) oligomer configurations sampled from a MD simulation in water, placing the lowest part of the oligomer at a random distance of between 4 and 9 Å above the surface. We then added a harmonic restraint (as typically used in NMR refinement) between the centre of the oligomer and the nearest atom of the surface, vertically below it and ran 20 ps of MD simulation at 298.15 K using the restraint. The restraint applied a harmonic force for distances 6 Å > $r$ > 9 Å using a force constant of 0.1 kcal $mol^{-1}$ Å$^{-2}$. This was followed by 20000 steps of energy minimisation. The surface was frozen for both MD and energy minimisation runs using the Amber belly dynamics. The final energy minimised configuration was then used to calculate a binding energy

$$E_{bind} = E_{complex} – E_{surface} - E_{molecule}$$

using the ALPB (Analytical Linearized Poisson-Boltmann)[13] approach to provide an implicit solvation energy as a replacement for an explicit solvent. Calculations were run using the hydrophobic cores of the **P1**, **P5** and **P7** polymers, together with the reference polymer **P8** polymer core. For each system we employed 1878 independent calculations to calculate a mean binding energy $\langle E_{bind} \rangle$.

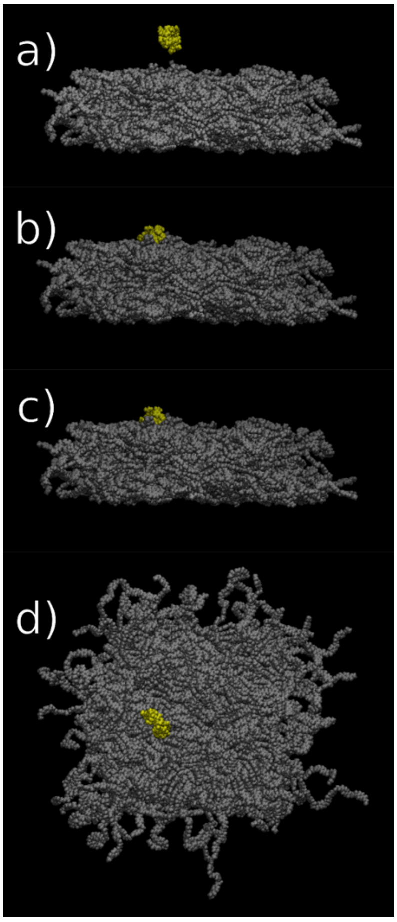


**Figure S6** Illustration of one cycle of the energy minimisation protocol. a) An independent configuration of the oligomer, taken from an MD calculation in water, is placed above the frozen PET surface. b) 20 ps of MD using NMR restraints is applied to bring the oligomer in contact with the surface. c) 20000 minimisation steps are carried out to give the structure shown. d) Plan view of the final structure. The binding energy of the final structure is obtained.

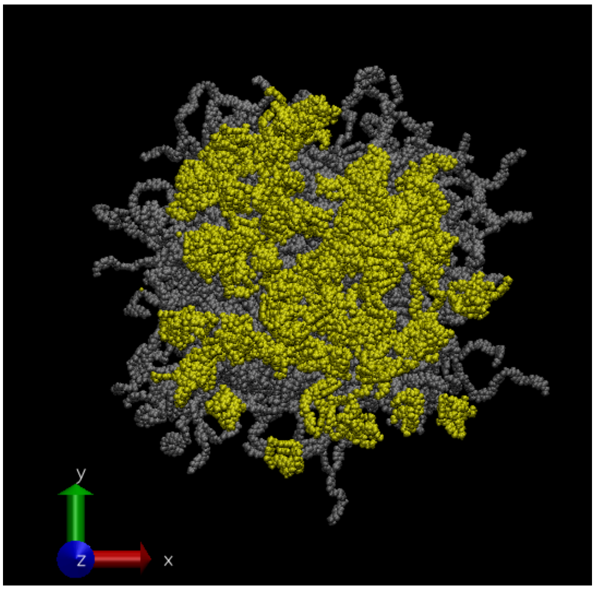


**Figure S7** Results from 112 separate independent energy minimisation calculations showing the sampling of the PET surface. The final results for <$E_{bind}$> were obtained from 1878 independent calculations.

## 7.5 Single polymer MD in water

### *7.5.1 Single polymer chains in water*

MD modelling of single chains in water show that the oligomer cores are characterized by folded structures, in which the flexible monomer linkers allow π-stacking to occur between aromatic groups, reminiscent of the relatively weak non-covalent aqueous solution stacking seen in chromonic liquid crystals (non-conventional amphiphiles).[14] The core is dynamic and on the timescale of the atomistic simulations is seen to partially unfold and refolds continuously, as shown below. Here, water acts as a reasonably good solvent for the PEG chains but a very poor solvent for the hydrophobic cores.

The PEG chains act to partially shield the core from water. However, as shown below, steric clashes arising from the 2,6-substitution lead to relatively poor folding of **P5** molecules with PEG chains sometimes passing through the core. This leads to an enlarged core structure which is less spherical than the core of **P1**, poorer shielding of core-water interactions and hence a larger driving force for aggregation of molecules in water. In **P9**, the shorter mPEG500 chains are similarly unable to shield the core from the surrounding aqueous environment, leading to increased driving force for aggregation.

### *7.5.2 Single P5 molecule in water*

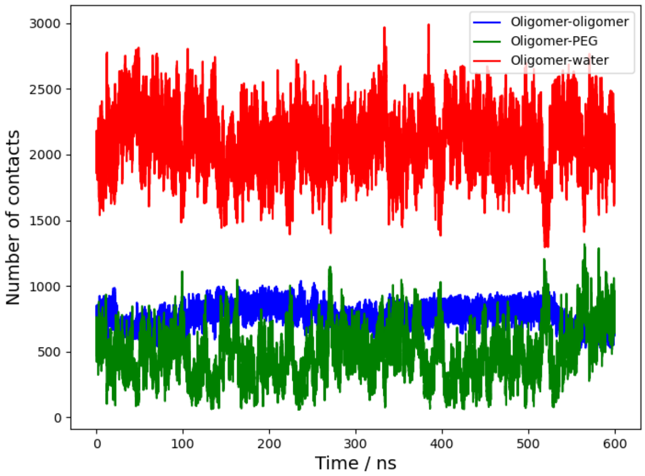


**Figure S8** Number of nonbonded interactions of the oligomer core for **P5** in water within a cutoff of 4.5 Å.

### *7.5.3 Single P1 molecule in water*

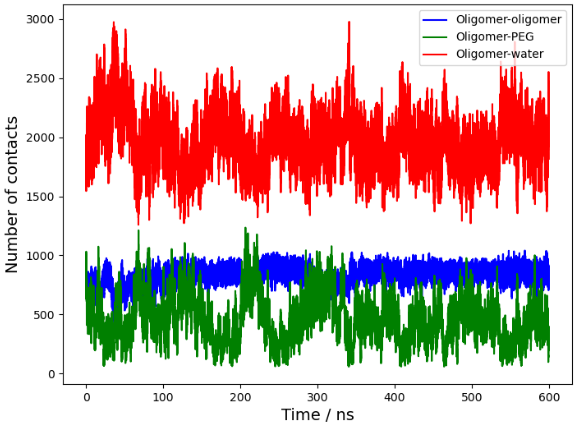


**Figure S9** Number of nonbonded interactions of the oligomer core for **P1** in water within a cutoff of 4.5 Å.

### *7.5.4 Single P9 molecule in water*

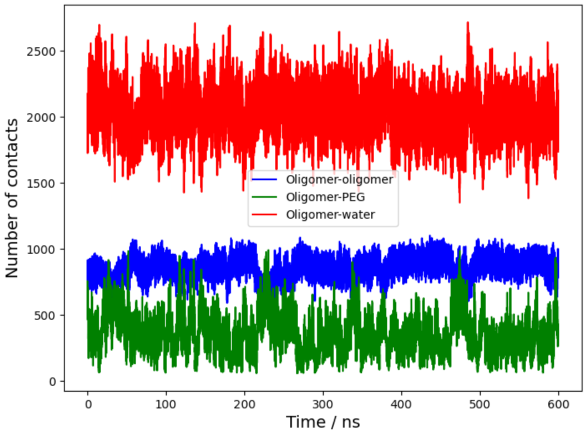


**Figure S10** Number of nonbonded interactions of the oligomer core for **P9** in water within a cutoff of 4.5 Å.

### *7.5.5 Comparison of the number of interactions between oligomer cores for single polymer molecules in water*

**Table S1** Number of interactions between oligomer cores within a 4.5 Å for single polymer molecules in water.

| **contacts** | **Reference SRP with short PEG chains** | **P8** | **P5 (2,6) polymer** | **P1 (2-5) polymer** |
|---|---|---|---|---|
| Oligomer-water | 2029 | 1872 | 2100 | 1963 |
| Oligomer-oligomer | 860 | 749 | 772 | 829 |
| Oligomer-PEG | 360 | 842 | 479 | 455 |

### *7.5.6 Comparison of the radius of gyration for P1 and P5 polymers in water.*

The steric clashes arising from 2,6 substitution leads to the hydrophobic core being less well-folded for **P5** in comparison to **P1**, as seen through a larger radius of gyration for the core and a larger eccentricity for the core moment of inertia spheroid.

**Table S2** Radius of gyration and eccentricity of the hydrophobic core calculated for **P5** and **P1** polymers in water.

| | $R_G$ for polymer / Å | $R_G$ for core / Å | Eccentricity, $e$ |
|---|---|---|---|
| **P5** | 20.1 ± 0.5 | 6.5 ± 0.1 | 0.63 |
| **P1** | 20.6 ± 0.8 | 6.2 ± 0.1 | 0.58 |

## 7.6 MD for ten polymer molecules in a water

### *7.6.1 Aggregation of P5 in a 10 %w/w solution*

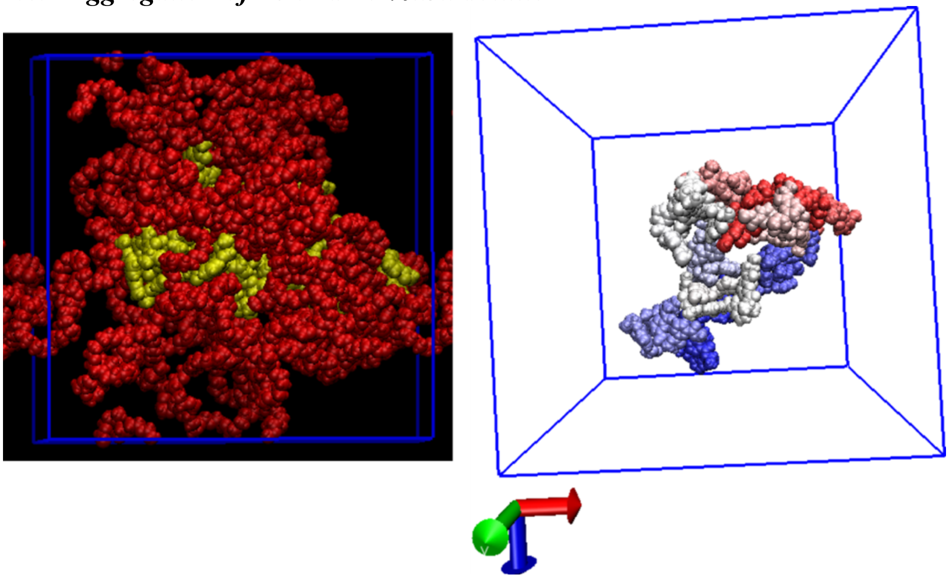

**Figure S11** Left - single aggregate of ten **P5** molecules after 355 ns of simulation in a 10% w/w solution. Right – hydrophobic cores of **P5**, shown for this aggregate.

### *7.6.2 Comparison of aggregation in solution for P1 and P5 in a 2 % w/w solution*

Solvated PEG chains partially shield the hydrophobic oligomer core from water. This has the effect of limiting aggregation in solution. However, the shape and size of the core have an effect on solution aggregation, as seen in the DLS results (Fig. 5, Fig. S4-5).

A comparison of the aggregation of **P5** and **P1** in 2% w/w (10 polymer molecules,144000 water molecules, 439970 sites) shows that **P5** has a much stronger driving force for aggregation initially due to a larger number of oligomer-water contacts resulting from the awkward folding of the core. Following the **P1** and **P5** solutions over 400 ns of simulation shows that the number of oligomer-water contacts evolve to a similar number in each system (~15000). However, over that period **P5** no longer has any monomers remaining, all molecules have formed aggregates and the poor shielding of the molecular cores in some of these aggregates suggest that these would grow further if a further supply of monomers was present. Whereas **P1** has stable monomers in equilibrium with some small aggregates. We note that **P5** shows a significantly larger number of oligomer-PEG interactions arising from the awkward folding of cores, which can transiently fold around a PEG chain for **P5**.

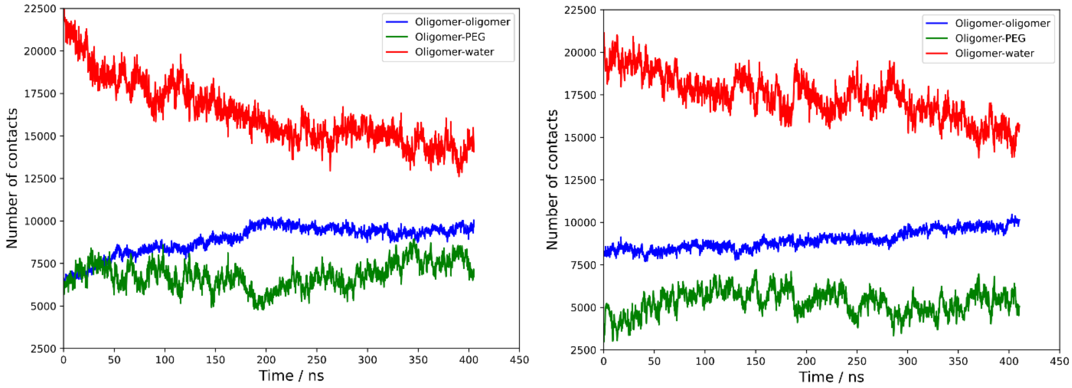


**Figure S12** Number of contacts for (left) **P5** and (right) **P1**. Calculations are carried out for 10 polymer molecules in 2% w/w solution.

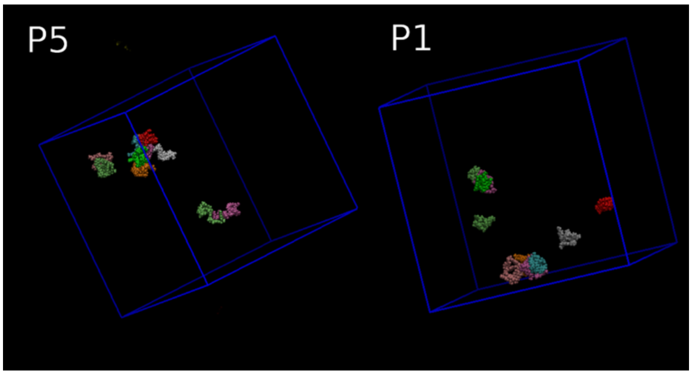


**Figure S13** Hydrophobic cores for 10 molecules of **P5** and **P1** in 2 % w/w solution after 400 ns of simulation. Individual cores are colour-coded. **P1** shows the presence of single molecules in solution but no unaggregated molecule remains for **P5**.

### *7.6.3 Aggregation of P9 in solution*

Atomistic molecular dynamics simulations of **P9** again suggested self- assembly of aromatic rings to form a folded core. PEG chains are oriented facing towards the surrounding environment, partially shielding the hydrophobic parts of the polymer from interactions with water (Figure S14a). The shorter mPEG500 chains are unable to sufficiently shield the hydrophobic core from the surrounding environment, however, supporting the observation of aggregates as observed by DLS. Simulation of the aggregation of **P9** in 2% w/w (10 polymer molecules, 65347 water molecules), demonstrated significant driving force for aggregation, with increasing oligomer-oligomer contacts and a concurrent decrease in oligomer-water contacts (Figure S14b).

**(a)** **(b)**

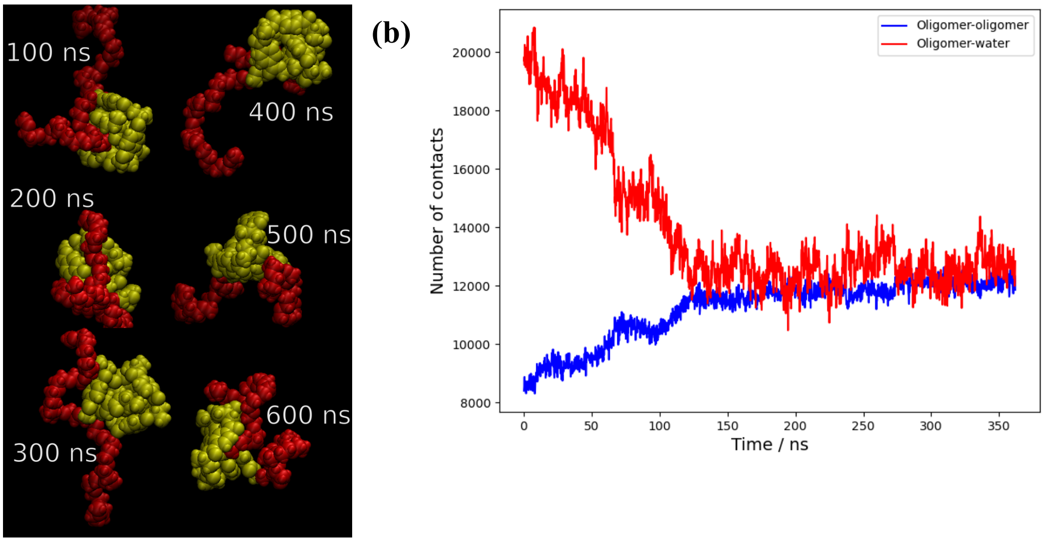


**Figure S14** (a)Time snapshots showing the folding and refolding of the hydrophobic core for **P9** in water over 600 ns, demonstrating that short PEG chains are unable to shield the hydrophobic core from the water, or protect it from aggregation with other molecules. (b) Number of contacts for **P9**. Calculations are carried out for 10 polymer molecules in 2% w/w solution.

### 7.7 Spontaneous adsorption of a reference SRP onto a PET surface

Figure S15 shows the capture of the terephthalate-based reference polymer **P8** by a polyester surface. Polymer capture is shown starting from the end of the equilibration run in which the PEG chains make first contact with the surface. Initially, the number of oligomer-surface contacts increases rapidly as the core settles onto the surface. This initial stage of capture occurs within the first 75 ns. This is followed by a slower process whereby the number of polymer-surface contacts slowly increases up to ~500 ns, during which the core slowly flattens onto the surface. During the course of the simulation the oligomer core remains folded as a self-assembled ball, while the PEG chains remain "unbound" and free to sample a large range of conformations close to the surface. Unwinding of the core is not seen on the timescale accessible to the atomistic simulations.

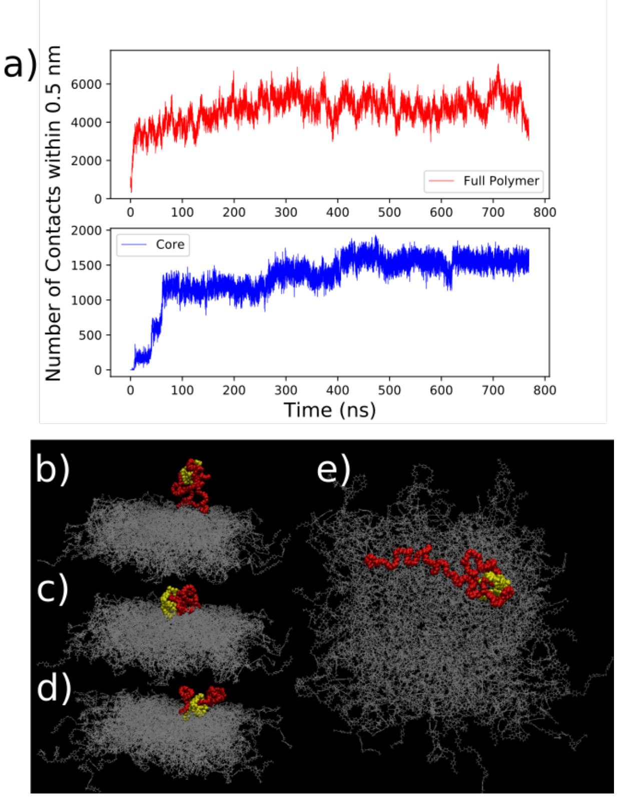


**Figure S15** Capture of **P8** by the polyester surface. a) Number of surface contacts for the oligomer core and the whole polymer as a function of time. b-d) Side view of the polymer at different simulation times after the initial 5 ns equilibration run. b) 0 ns, c) 50 ns, d) 770 ns. e) Plan view after 770 ns. The oligomer core is shown in yellow and PEG chains are shown in red.

### 7.8 Binding energies

From the MD calculations of §7.7 it is believed that the folded core is favourably attracted to the PET surface, and likely interacts quite strongly without necessarily unwinding. The strength of interaction for different cores can be made by considering only the hydrophobic core and a series of energy minimisation calculations to average over the different atomic environments of the surface. The histograms shown in Figure S16 represent the results of these calculations using the protocol described in §7.4, which involves 1878 independent energy minimisations per system.

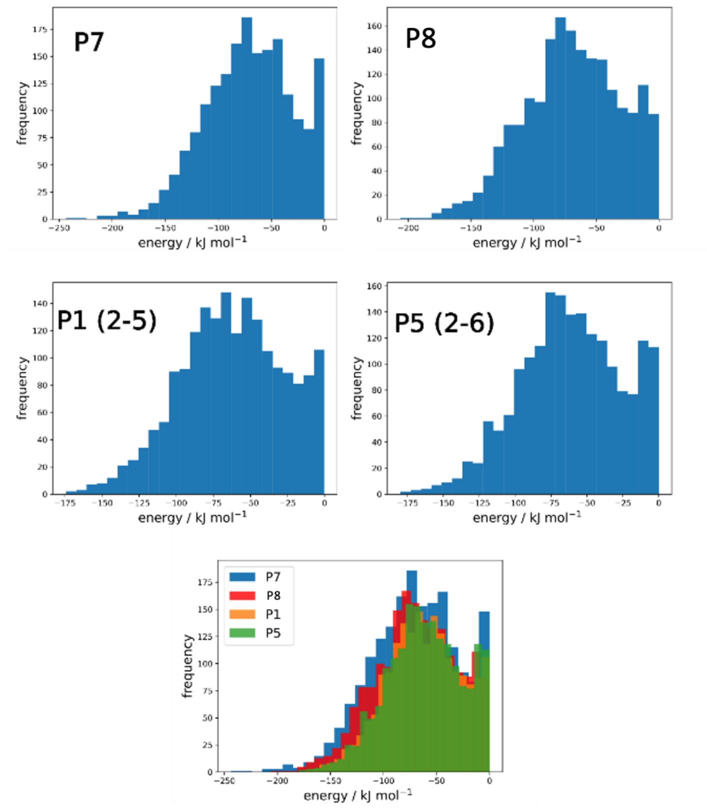


**Figure S16** Histograms of binding energies, $E_{\text{bind}}$, obtained from 1878 independent minimisation calculations.

### 7.9 Quantum chemical calculations of torsional energy barriers

Quantum chemical calculations were carried out at the MP2/6-31+g(d,p) level to obtain torsional energy barriers to internal rotational within the polymers to both parametrise the force field used and assess the internal flexibility of the different polymer chains.

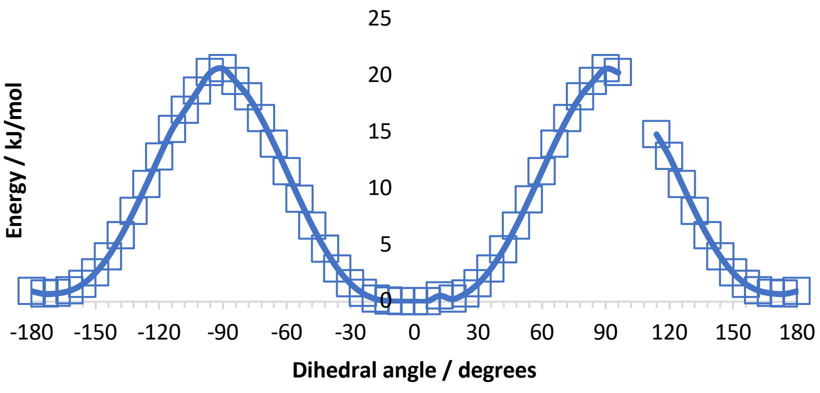


**Figure S17** MP2/6-31+g(d,p) dihedral angle energy from relaxed scan for the C(ar)-C(ar)-C(sp2)-O(sp3) dihedral angle in dimethyl terephthalate

**Table S3** Conformational energies of dimethyl terephthalate.

| **Conformation** | Relative energy / kJ $mol^{-1}$ |
| --- | --- |
| 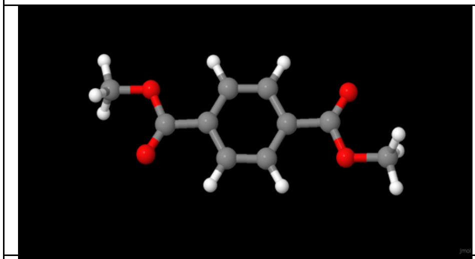 | 0.0 |
| 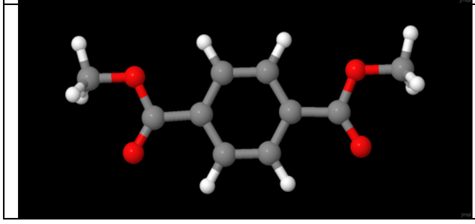 | 0.68 |

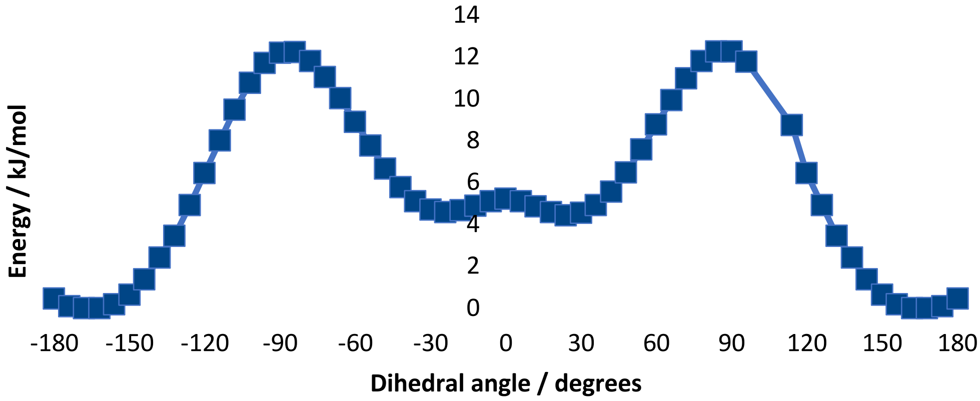


**Figure S18** MP2/6-31+g(d,p) dihedral angle energy from relaxed scan for the C(ar)-C(ar)-C(sp2)-O(sp3) dihedral angle in dimethyl pyridine-2,6-dicarboxylate.

**Table S4** Conformational energies of dimethyl pyridine-2,6-dicarboxylate.

| **Conformation** | Relative energy / kJ $mol^{-1}$ |
| --- | --- |
| 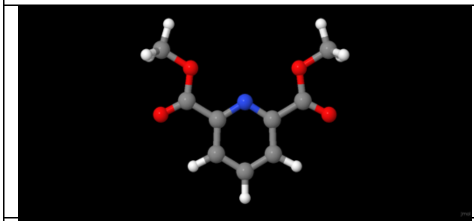 | 0.0 |
| 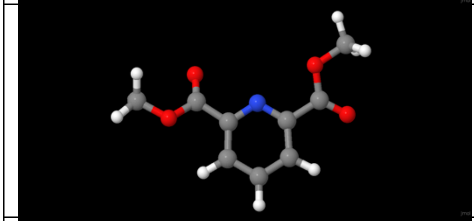 | 4.0 |
| 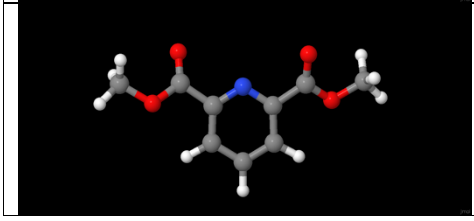 | 9.4 |

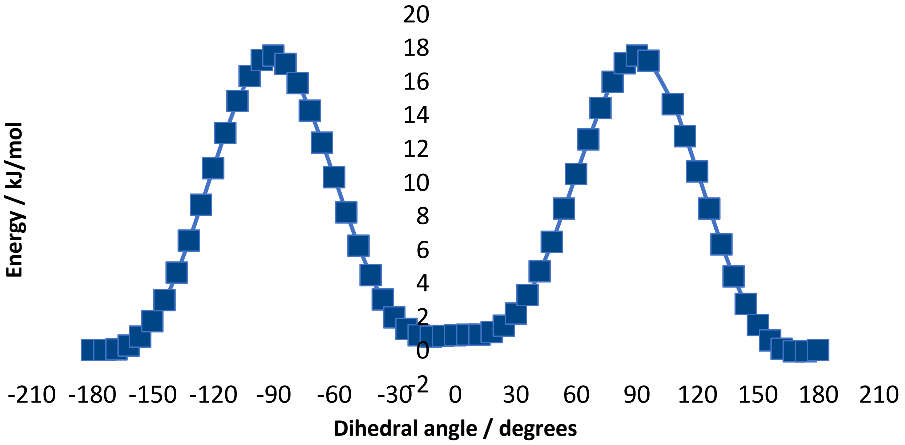


**Figure S19** MP2/6-31+g(d,p) dihedral angle energy from relaxed scan for the C(ar)-C(ar)-C(sp2)-O(sp3) dihedral angle in dimethyl pyridine-2,5-dicarboxylate.

**Table S5** Conformational energies of dimethyl pyridine-2,5-dicarboxylate.

| **Conformation** | Relative energy / kJ $mol^{-1}$ |
|---|---|
| 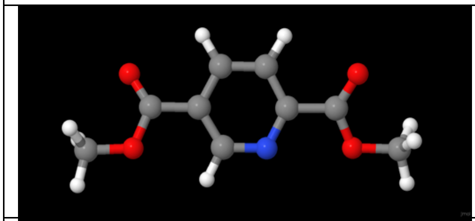 | 0.0 |
| 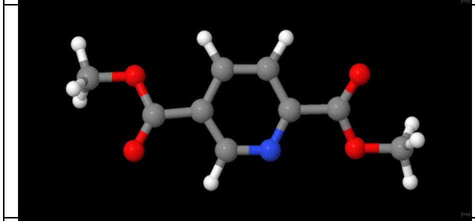 | 0.89 |
| 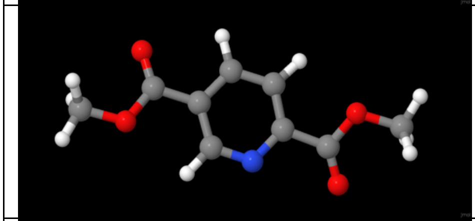 | 4.2 |
| 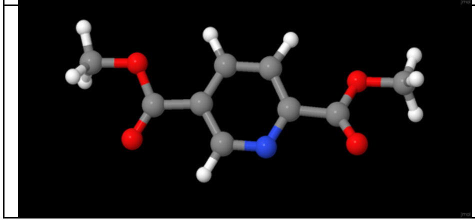 | 5.6 |

### 7.10 Effects of isomerism within pyridine dicarboxylate unit on aggregation

**P1** (2,5-isomer) and **P5** (2,6-isomer) display markedly different behaviour in solution. The *meta*- ester groups in **P5** give rise to steric clashes within the hydrophobic core, leading to poorer π-stacking in **P5** compared to **P1**. This effect can be seen quite clearly by plotting the distribution function of the distances of the first atoms in the PEG chains (i.e. the two atoms immediately adjacent to the hydrophobic core; Figure S20).

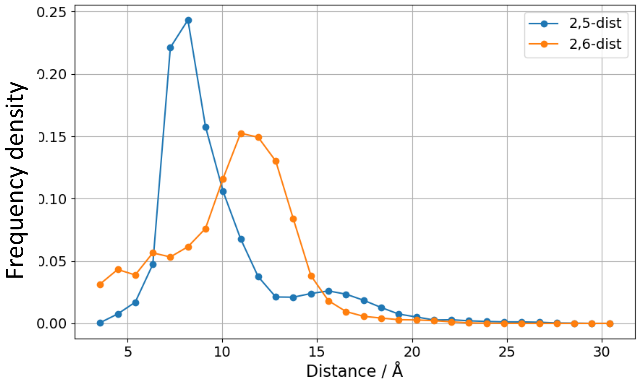


**Figure S20** Normalized distance distribution functions for the distance between the first atoms in the two PEG chains for **P1** and **P5**. Distribution functions are calculated for single polymer molecules in water over ~ 600 ns.

The strong peak at relatively short distances for **P1** arises from a well-folded structure with π-stacking. **P5** is less well-folded, leading to a less spherical core and to greater conformational freedom. This can be quantified by calculating the eccentricity, e, associated with the moment of inertia of the hydrophobic core (noting that e = 0, represents a perfect sphere and e = 1 represents a rod or disk).

**Table S6:** Eccentricity, *e*, of the moment of inertia of the hydrophobic cores for three polymers, calculated for a single polymer chain in water.

| **Polymer** | *e* |
|---|---|
| **P1** | 0.579+/- 0.009 |
| **P5** | 0.634 +/- 0.006 |
| **P8** | 0.606 +/-0.007 |

The less spherical core for **P5** leads to its decreased shielding by the PEG chains from water interactions .This is demonstrated numerically by the number of oligomer core–water interactions for **P1** and **P5** and the reference polymer **P8** (Table S1). A simple visual comparison can also be demonstrated by calculating the (Jacobian normalized) angle distribution between the bond vectors at the end of the hydrophobic core (Figure S21). For **P1** the vectors are mainly pointing in opposite directions indicating that the PEG chains are pointing out from opposite sides of the folded structure. However, for **P5**, the conformationally fluctuating core leads to no strong preference for the direction the PEG chains point out from the core, and so these chains are often pointing out in the same direction providing less shielding of the hydrophobic core from interactions with water, and hence a greater driving force for aggregation.

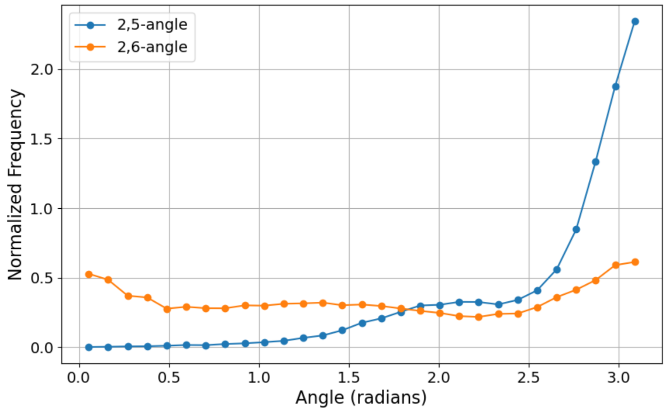


**Figure S21**: Distribution of angles for bond vectors at the ends of the hydrophobic core. Distribution functions are calculated for single polymer molecules **P1** and **P5** in water over ~ 600 ns.

## 8. NMR spectra

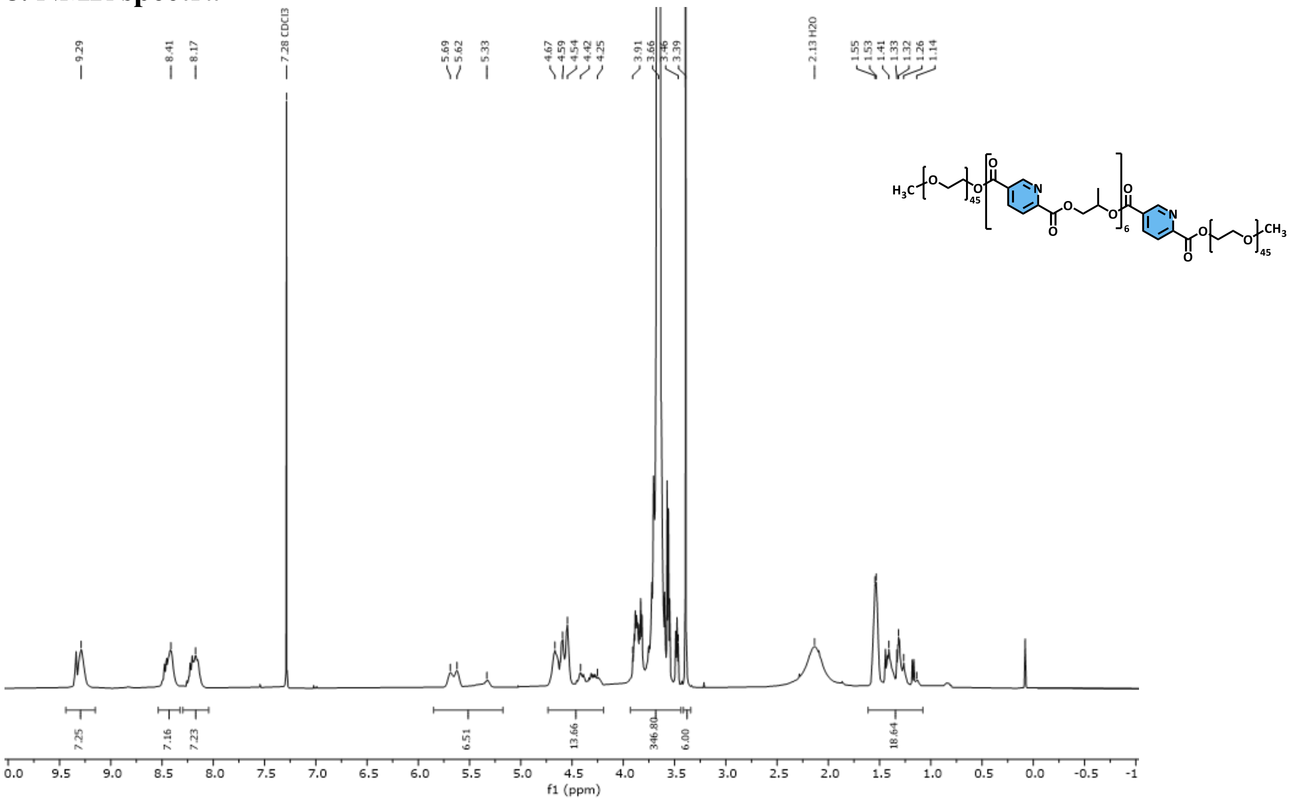


**Figure S22** $^{1}H$ NMR ($CDCl_3$, 400 MHz) of **P1**

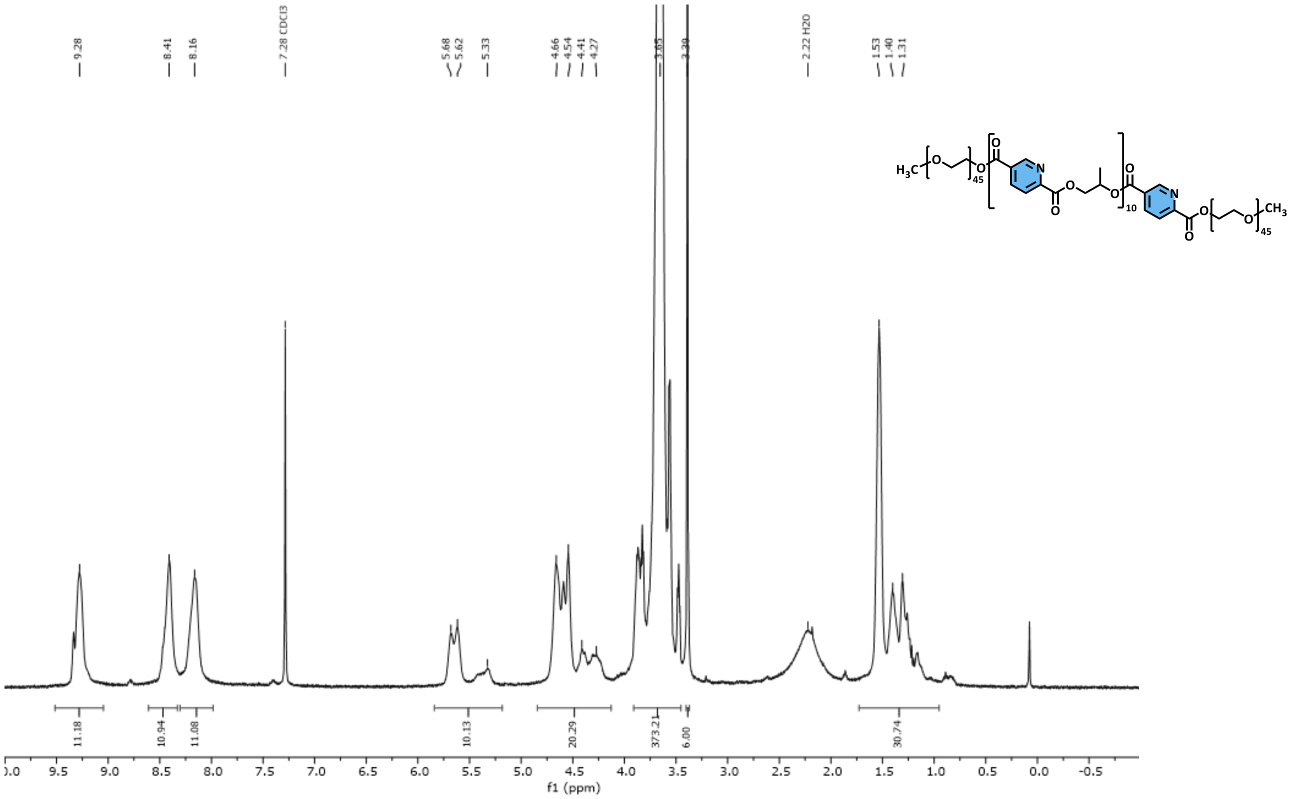


**Figure S23** $^{1}H$ NMR ($CDCl_3$, 400 MHz) of **P2**

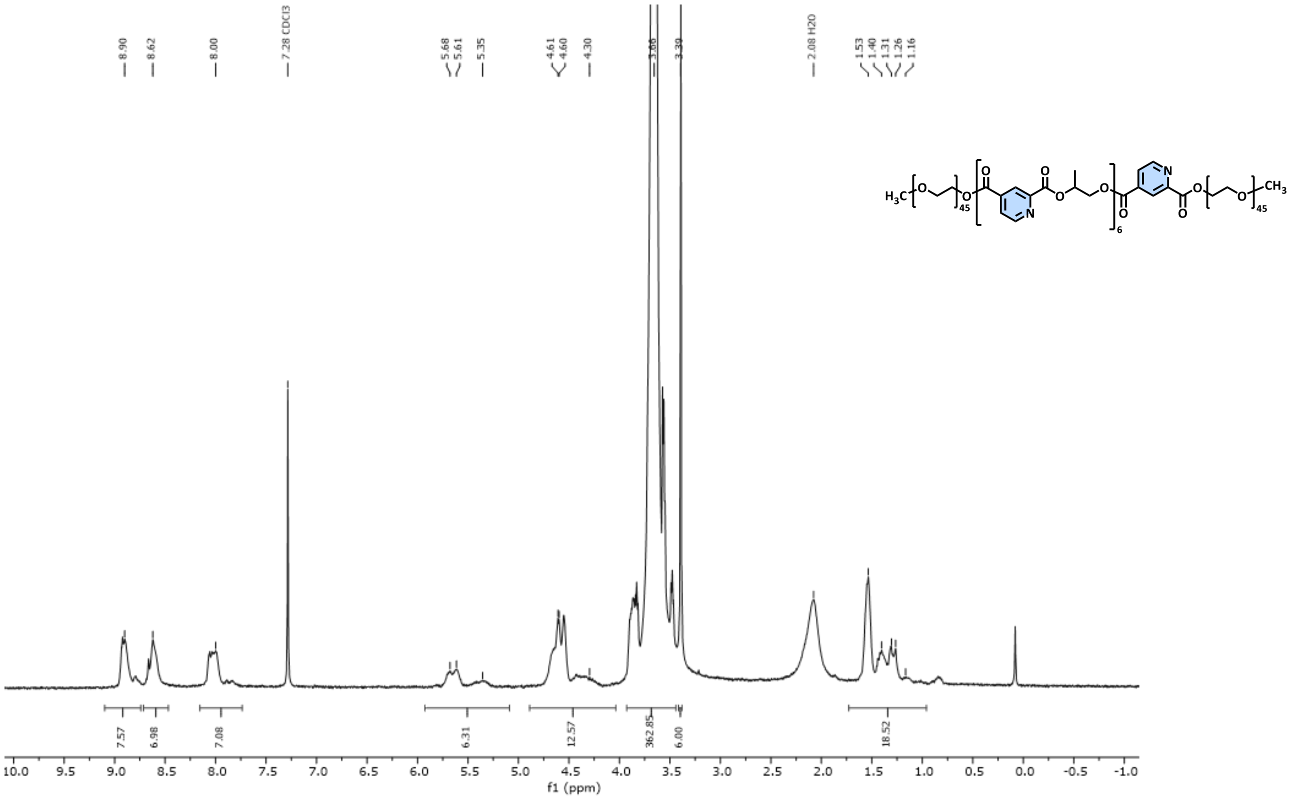


**Figure S24** $^{1}$H NMR ($CDCl_3$, 400 MHz) of **P3**

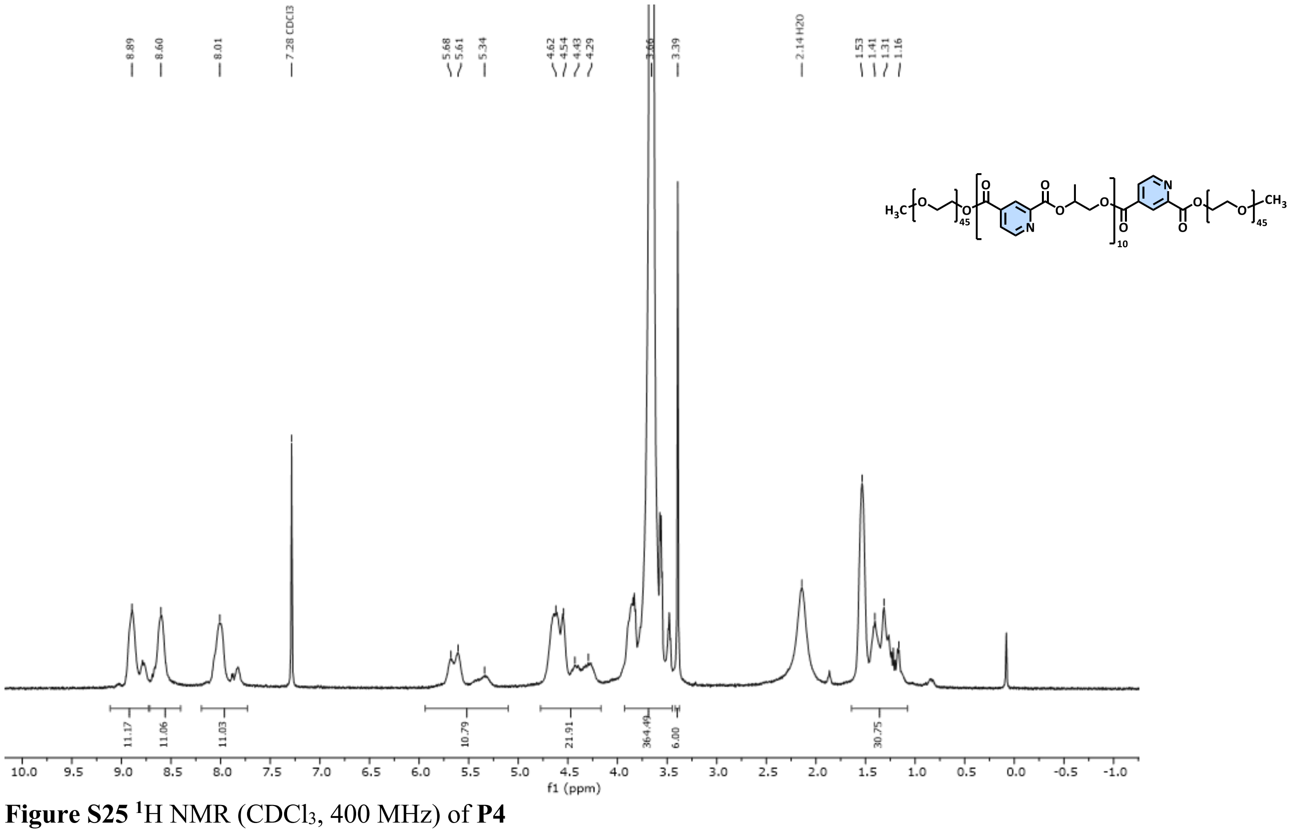


**Figure S25** $^{1}$**H** NMR ($CDCl_3$, 400 MHz) of **P4**

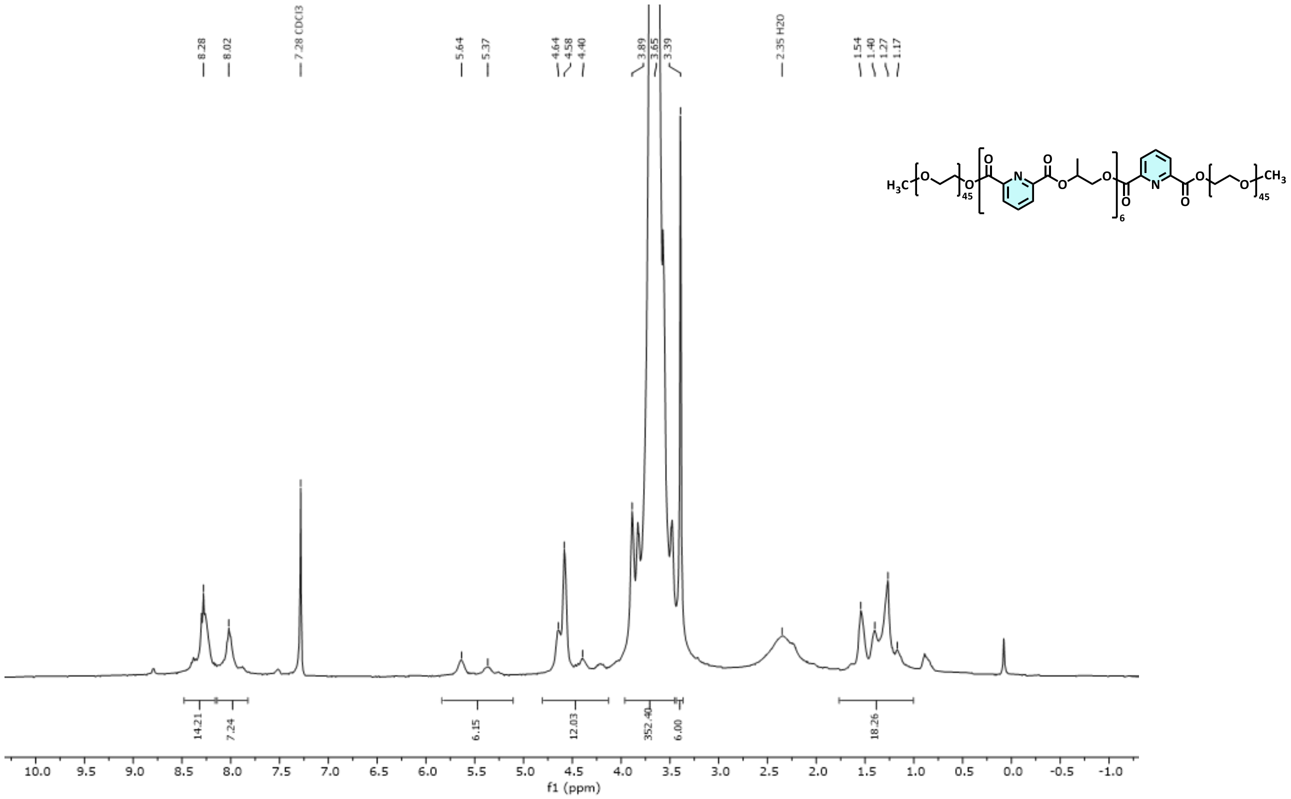

**Figure S26** $^1$H NMR ($CDCl_3$, 400 MHz) of **P5**

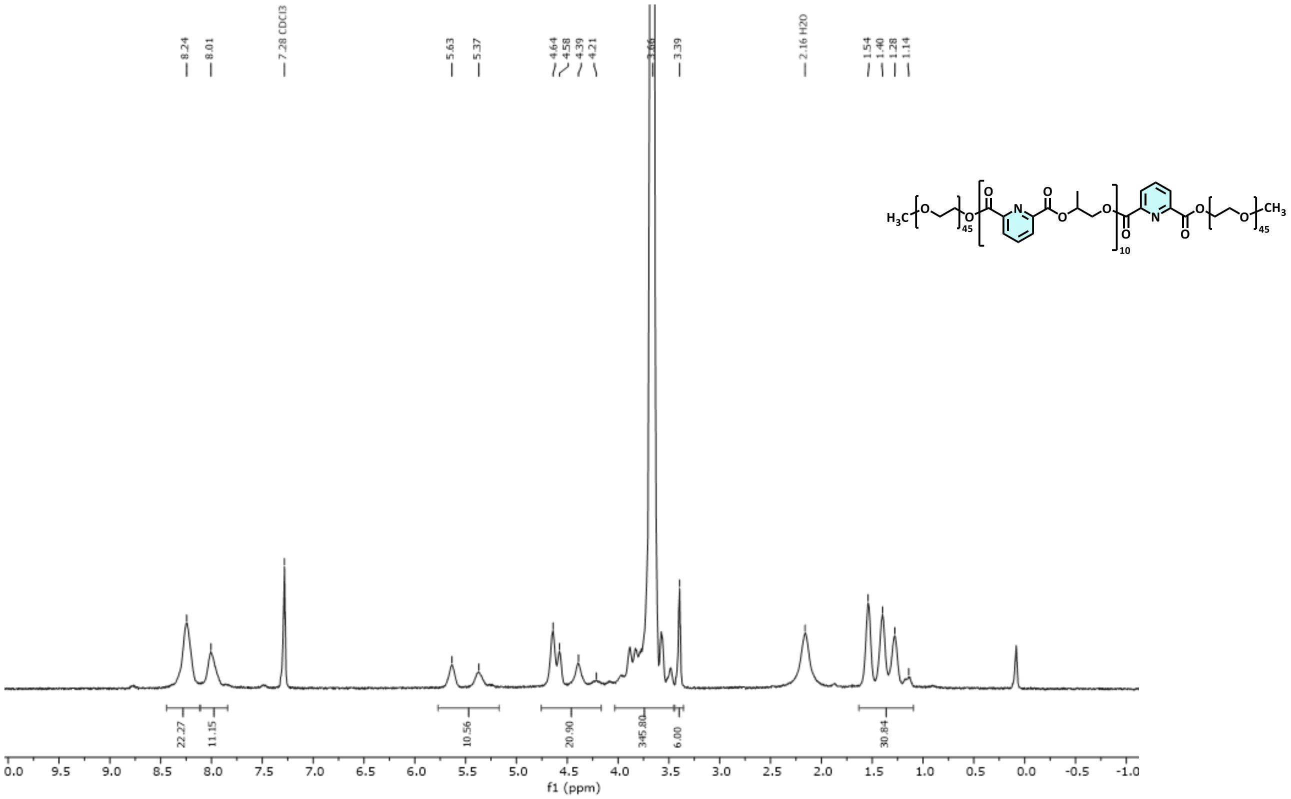

**Figure S27** $^1$H NMR ($CDCl_3$, 400 MHz) of **P6**

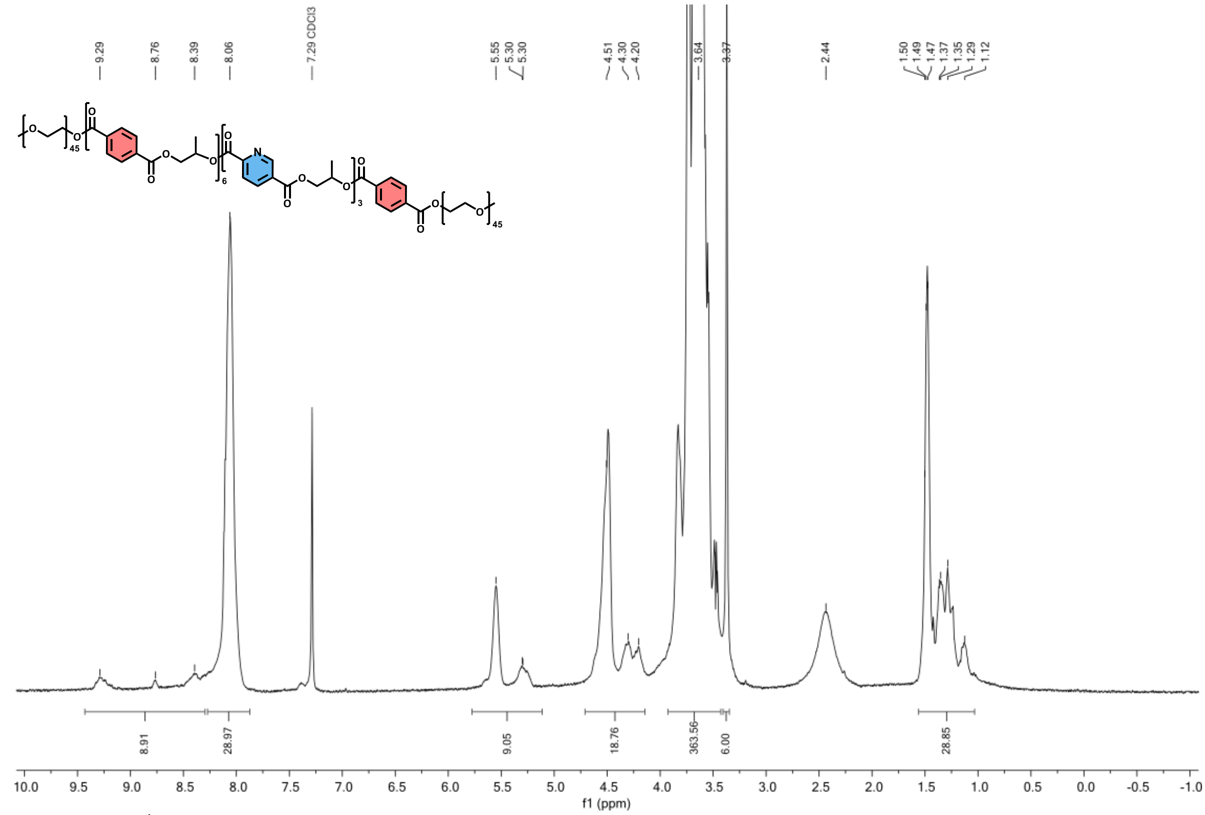

**Figure S28** $^1$H NMR ($CDCl_3$, 400 MHz) of **P7**

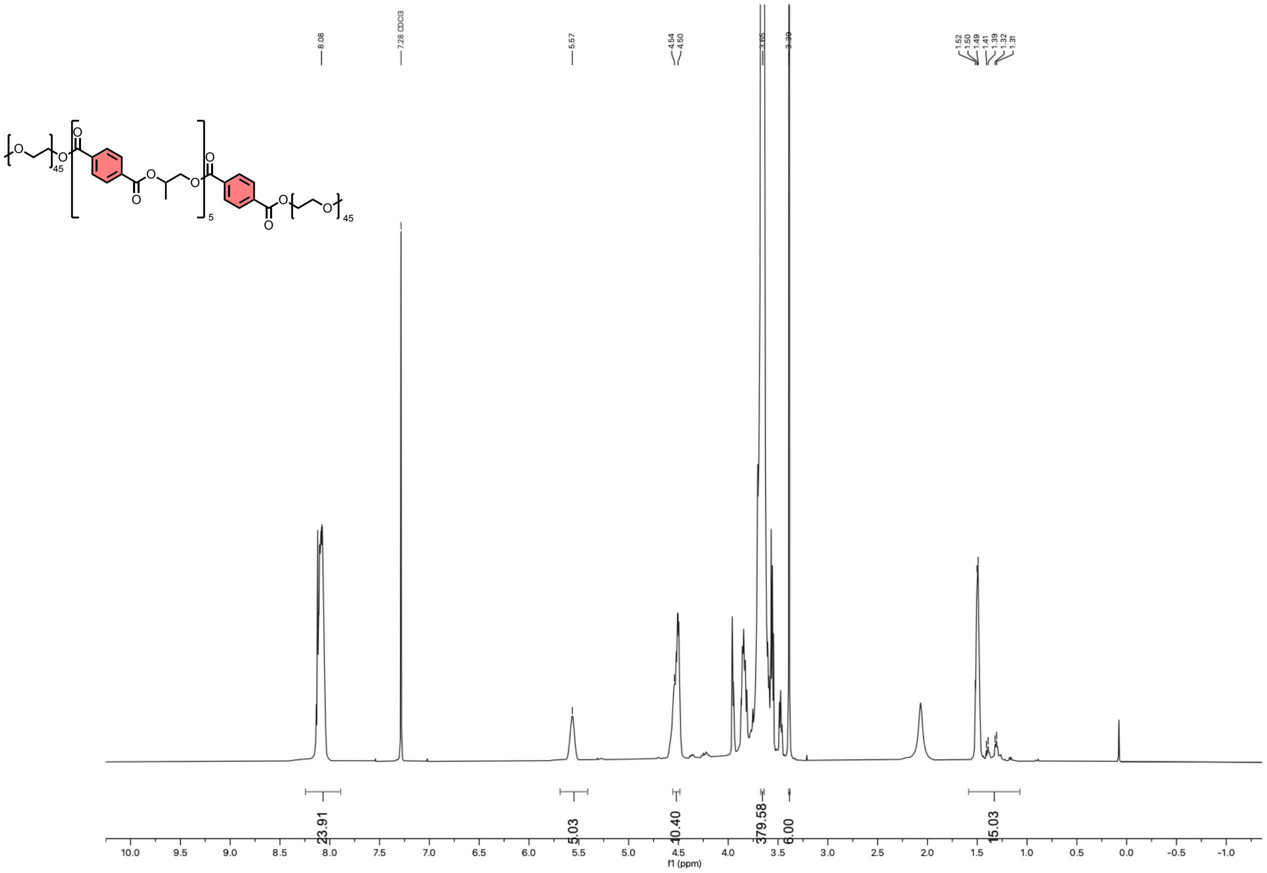

**Figure S29** $^1$H NMR ($CDCl_3$, 400 MHz) of **P8**

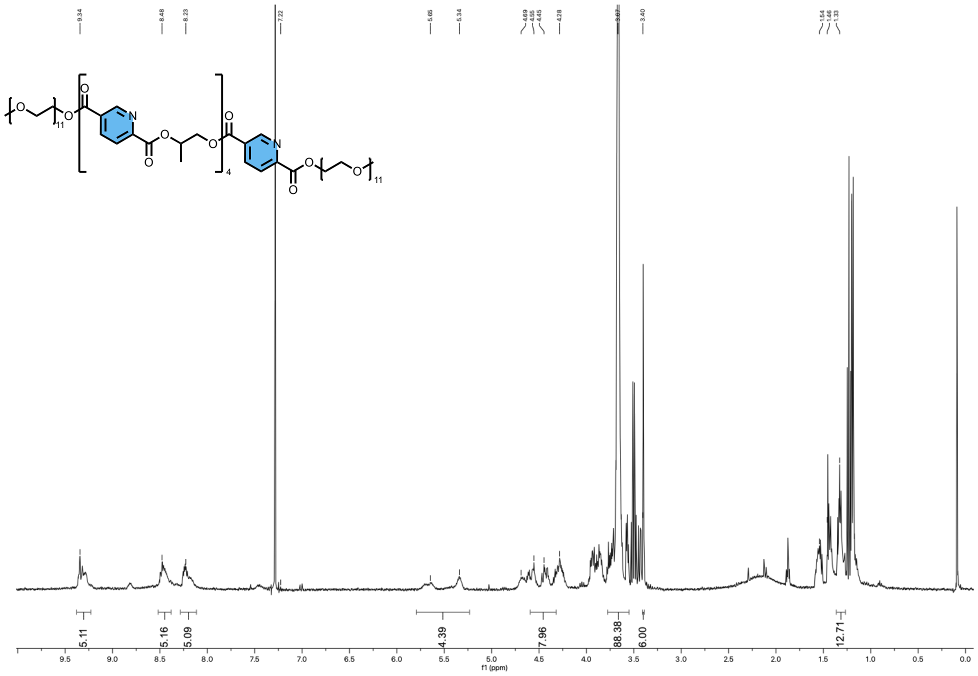

**Figure S30** $^{1}$H NMR ($CDCl_3$, 400 MHz) of **P9**

## References


(1) Case, D. A.; Aktulga, H. M.; Belfon, K.; Cerutti, D. S.; Cisneros, G. A.; Cruzeiro, V. W. D.; Forouzesh, N.; Giese, T. J.; Götz, A. W.; Gohlke, H.; et al. AmberTools. *J. Chem. Inf. Model.* **2023**, *63* (20), 6183-6191. DOI: 10.1021/acs.jcim.3c01153 (acccessed 2024/02/16/16:56:17). From ACS Publications.

(2) Jakalian, A.; Jack, D. B.; Bayly, C. I. Fast, efficient generation of high-quality atomic charges. AM1-BCC model: II. Parameterization and validation. *J. Comput. Chem.* **2002**, *23* (16), 1623-1641. DOI: 10.1002/jcc.10128.

(3) Jakalian, A.; Bush, B. L.; Jack, D. B.; Bayly, C. I. Fast, efficient generation of high-quality atomic Charges. AM1-BCC model: I. Method. *J. Comput. Chem.* **2000**, *21* (2), 132-146. DOI: Doi 10.1002/(Sici)1096-987x(20000130)21:2<132::Aid-Jcc5>3.3.Co;2-G.

(4) Wang, J.; Wolf, R. M.; Caldwell, J. W.; Kollman, P. A.; Case, D. A. Development and testing of a general amber force field. *J. Comput. Chem.* **2004**, *25* (9), 1157-1174. DOI: 10.1002/jcc.20035 (acccessed 2018-05-14 16:29:25).Wiley Online Library.

(5) Boyd, N. J.; Wilson, M. R. Optimization of the GAFF force field to describe liquid crystal molecules: the path to a dramatic improvement in transition temperature predictions. *Phys. Chem. Chem. Phys.* **2015**, *17* (38), 24851-24865. DOI: 10.1039/c5cp03702f. Boyd, N. J.; Wilson, M. R. Validating an optimized GAFF force field for liquid crystals: T-NI predictions for bent-core mesogens and the first atomistic predictions of a dark conglomerate phase. *Phys. Chem. Chem. Phys.* **2018**, *20* (3), 1485-1496. DOI: 10.1039/c7cp07496d.

(6) *Gaussian 16 Rev. C.01*; Wallingford, CT, 2016. (accessed.

(7) Barbosa, N. S. V.; Zhang, Y.; Lima, E. R. A.; Tavares, F. W.; Maginn, E. J. Development of an AMBER-compatible transferable force field for poly(ethylene glycol) ethers (glymes). *J Mol. Model.* **2017**, *23* (6), 194. DOI: 10.1007/s00894-017-3355-3.

(8) Ottallah, T.; Parandian, S. A.; Rick, S. W. Analysis of Atomistic Potentials for Poly(ethylene glycol) Ethers. *J. Chem. Theory Comput.* **2021**, *17* (1), 315-321. DOI: 10.1021/acs.jctc.0c00871.

(9) Fischer, J.; Paschek, D.; Geiger, A.; Sadowski, G. Modeling of aqueous poly(oxyethylene) solutions: 1. Atomistic simulations. *J. Phys. Chem. B* **2008**, *112* (8), 2388-2398. DOI: 10.1021/jp0765345.

(10) Kawaguchi, S.; Imai, G.; Suzuki, J.; Miyahara, A.; Kitano, T.; Ito, K. Aqueous solution properties of oligo- and poly(ethylene oxide) by static light scattering and intrinsic viscosity. *Polymer* **1997**, *38* (12), 2885-2891. DOI: 10.1016/s0032-3861(96)00859-2.
(11) Sherck, N.; Webber, T.; Brown, D. R.; Keller, T.; Barry, M.; DeStefano, A.; Jiao, S.; Segalman, R. A.; Fredrickson, G. H.; Shell, M. S.; et al. End-to-End Distance Probability Distributions of Dilute Poly(ethylene oxide) in Aqueous Solution. *J. Am. Chem. Soc.* **2020**, *142* (46), 19631-19641. DOI: 10.1021/jacs.0c08709.
(12) D.A. Case, H. M. A., K. Belfon, I.Y. Ben-Shalom, J.T. Berryman, S.R. Brozell, D.S. Cerutti, T.E. Cheatham, III, G.A. Cisneros, V.W.D. Cruzeiro, T.A. Darden, N. Forouzesh, G. Giambaşu, T. Giese, M.K. Gilson, H. Gohlke, A.W. Goetz, J. Harris, S. Izadi, S.A. Izmailov, K. Kasavajhala, M.C. Kaymak, E. King, A. Kovalenko, T. Kurtzman, T.S. Lee, P. Li, C. Lin, J. Liu, T. Luchko, R. Luo, M. Machado, V. Man, M. Manathunga, K.M. Merz, Y. Miao, O. Mikhailovskii, G. Monard, H. Nguyen, K.A. O'Hearn, A. Onufriev, F. Pan, S. Pantano, R. Qi, A. Rahnamoun, D.R. Roe, A. Roitberg, C. Sagui, S. Schott-Verdugo, A. Shajan, J. Shen, C.L. Simmerling, N.R. Skrynnikov, J. Smith, J. Swails, R.C. Walker, J. Wang, J. Wang, H. Wei, X. Wu, Y. Wu, Y. Xiong, Y. Xue, D.M. York, S. Zhao, Q. Zhu, and P.A. Kollman Amber 2023, University of California, San Francisco. 2023.
(13) Sigalov, G.; Fenley, A.; Onufriev, A. Analytical electrostatics for biomolecules: Beyond the generalized Born approximation. *J. Chem. Phys.* **2006/03/28**, *124* (12). DOI: 10.1063/1.2177251. Sigalov, G.; Scheffel, P.; Onufriev, A. Incorporating variable dielectric environments into the generalized Born model. *J. Chem. Phys.* **2005/03/01**, *122* (9). DOI: 10.1063/1.1857811.
(14) Yu, G.; Wilson, M. R. Molecular simulation studies of self-assembly for a chromonic perylene dye: All-atom studies and new approaches to coarse-graining. *J. Mol. Liq.* **2022**, *345*. DOI: ARTN 118210
10.1016/j.molliq.2021.118210. Chami, F.; Wilson, M. R. Molecular order in a chromonic liquid crystal: a molecular simulation study of the anionic azo dye sunset yellow. *J. Am. Chem. Soc.* **2010**, *132* (22), 7794-7802. DOI: 10.1021/ja102468g. Yu, G.; Walker, M.; Wilson, M. R. Atomistic simulation studies of ionic cyanine dyes: self-assembly and aggregate formation in aqueous solution. *Phys. Chem.Chem. Phys.* **2021**, *23* (11), 6408-6421. DOI: 10.1039/d0cp06205g.
